# SPRTN patient variants cause global-genome DNA-protein crosslink repair defects

**DOI:** 10.1038/s41467-023-35988-1

**Published:** 2023-01-21

**Authors:** Pedro Weickert, Hao-Yi Li, Maximilian J. Götz, Sophie Dürauer, Denitsa Yaneva, Shubo Zhao, Jacqueline Cordes, Aleida C. Acampora, Ignasi Forne, Axel Imhof, Julian Stingele

**Affiliations:** 1 Department of Biochemistry, Ludwig-Maximilians-University, 81377 Munich, Germany; 2 Gene Center, Ludwig-Maximilians-University, 81377 Munich, Germany; 3 Protein Analysis Unit (ZfP), BioMedical Center (BMC), Ludwig-Maximilians-University, 82152 Martinsried, Germany

**Keywords:** DNA damage and repair, Proteolysis, Biological techniques

## Abstract

DNA-protein crosslinks (DPCs) are pervasive DNA lesions that are induced by reactive metabolites and various chemotherapeutic agents. Here, we develop a technique for the Purification of x-linked Proteins (PxP), which allows identification and tracking of diverse DPCs in mammalian cells. Using PxP, we investigate DPC repair in cells genetically-engineered to express variants of the SPRTN protease that cause premature ageing and early-onset liver cancer in Ruijs-Aalfs syndrome patients. We find an unexpected role for SPRTN in global-genome DPC repair, that does not rely on replication-coupled detection of the lesion. Mechanistically, we demonstrate that replication-independent DPC cleavage by SPRTN requires SUMO-targeted ubiquitylation of the protein adduct and occurs in addition to proteasomal DPC degradation. Defective ubiquitin binding of SPRTN patient variants compromises global-genome DPC repair and causes synthetic lethality in combination with a reduction in proteasomal DPC repair capacity.

## Abstract (web summary)

DNA-protein crosslinks (DPCs) are toxic DNA lesions which threaten genome stability. Here, the authors develop a method to track the fate of DPCs in cells and identify a role for the SPRTN protease in replication-independent DPC repair.

## Introduction

Unrepaired DNA damage causes ageing and cancer formation^1,2^. Therefore, cells employ DNA repair pathways, which operate not only in a transcription-^3^ or replication-coupled^4^ manner, but also involve global mechanisms that scan the entire genome for lesions^5^. Covalent DNA-protein crosslinks (DPCs) are a particular pervasive type of DNA damage and are targeted by multiple repair enzymes^6^. DNA-protein crosslinking arises from enzymatic and non-enzymatic sources^7^. Non-enzymatic DPC formation is induced by bifunctional chemical crosslinkers such as platinum-based chemotherapeutics or formaldehyde, which is even produced within chromatin during histone demethylation and is present at micromolar concentrations in mammalian blood^8^. Enzymatic DPCs are caused by entrapment of normally transient covalent enzyme-DNA reaction intermediates and are induced by various chemotherapeutic agents including topoisomerase poisons and the antineoplastic drug 5-aza-2′-deoxycytidine (5-azadC)^9^. 5-azadC is incorporated into DNA during replication, where it acts as pseudo-substrate for DNA methyltransferase 1 (DNMT1) leading to formation of a covalent complex between the modified base and DNMT1’s active site cysteine^10,11^. However, upon methylating 5-azadC, DNMT1 fails to release from DNA, thereby forming a stable DPC.

DPC repair involves the proteolytic degradation of the protein adduct by metalloproteases of the Wss1/SPRTN family^12–17^. While loss of SPRTN is lethal in mammalian cells, hypomorphic variants cause Ruijs-Aalfs syndrome, which is characterized by premature ageing and early-onset hepatocellular carcinoma^16,18,19^. Ruijs-Aalfs syndrome is primarily caused by frame-shift mutations resulting in expression of C-terminally truncated SPRTN-ΔC variants, which lack nuclear localisation signals and various protein-protein interaction motifs^18^. Data obtained in frog egg extracts demonstrated that DPC cleavage by SPRTN can be initiated by a replication fork colliding with a DPC^20–22^. While the replicative helicase is able to bypass the protein adduct, DNA polymerases fail to synthesize across the DPC^20,21^. SPRTN recognizes the resulting single-/double-stranded DNA junction using a bipartite DNA-binding module, which triggers local activation of the enzyme and concurrent DPC cleavage^23^. In egg extracts, DPCs are additionally targeted by replication-coupled proteasomal degradation^20^. Recent reports suggest that the proteasome also targets DPCs outside of replication, which relies on initial SUMOylation of the protein adduct and subsequent ubiquitylation by the SUMO-targeted ubiquitin ligase RNF4^24,25^. In contrast, it is currently believed that SPRTN acts exclusively at the replication fork, where it relies on ubiquitin signals for recruitment^20,26^. No consensus has emerged regarding the role of SUMO modifications for SPRTN-dependent DPC repair. SUMOylation has been suggested to block alternative repair pathways to promote SPRTN-dependent repair^26,27^; while SUMOylation was found to be dispensable for SPRTN function in another study^28^. At any rate, the existence of at least two proteolytic systems to degrade DPCs - SPRTN and the proteasome - indicates significant evolutionary pressure to cope with these insults in order to preserve genome integrity. However, the relationship between proteasome- and SPRTN-dependent repair as well as their relative contribution towards DPC cleavage in mammalian cells remain unknown.

Exploring DPC repair in mammalian cells in mechanistic detail has remained challenging not only due to the essential function of the SPRTN protease, but also due to limitations of the currently available techniques for the study of DPCs. DPC formation in mammalian cells can be assessed by separating DPCs from non-crosslinked proteins using ultra-centrifugation of caesium chloride gradients^29,30^. However, this approach is laborious, low throughput, and requires substantial amounts of material. Most other DPC assays are based on precipitation as the separating principle. In the KCl-SDS assay (and its derivative ARK)^31,32^, proteins are precipitated from denaturing lysates and co-precipitating DNA is quantified as a proxy for the amount of DPCs. The RADAR (rapid approach to DNA adduct recovery) assay employs the opposite principle; DNA is precipitated from lysates and co-precipitating proteins are analysed using slot-blotting or silver-staining^33^. The reliance on precipitation is a major drawback of these assays. DPCs are diverse in nature, which will affect their behaviour during precipitation. While smaller protein adducts may efficiently co-precipitate with DNA, larger adducts may even prevent DNA from precipitating.

Here, we present a method for the Purification of x-linked Proteins (PxP) that overcomes these limitations. PxP is based on electro-elution of non-covalently attached proteins from DNA embedded in agarose plugs and can be combined with label-free quantitative mass spectrometry to determine the identity of unknown DPCs. In addition, we developed genetically-engineered hypomorphic *SPRTN* mutant cell lines expressing patient-mimicking variants enabling not only structure-function analysis of SPRTN in cells, but also the genetic exploration of relationships between different DPC repair factors. Using these tools, we describe an unexpected role for SPRTN in replication-independent DPC repair. We find that this global-genome DPC cleavage by SPRTN requires SUMO-targeted ubiquitylation of the DPC, occurs independent of proteasomal degradation, and is defective in cells expressing Ruijs-Aalfs syndrome-associated SPRTN variants. As a consequence, reduction of proteasomal DPC degradation causes synthetic defects in *SPRTN* mutant cell lines. Finally, structure-function analysis of SPRTN demonstrates that the loss of a ubiquitin-binding domain in patient variants is responsible for defective global-genome DPC repair.

## Results

### A strategy for the purification of crosslinked proteins

**Fig. 1:**
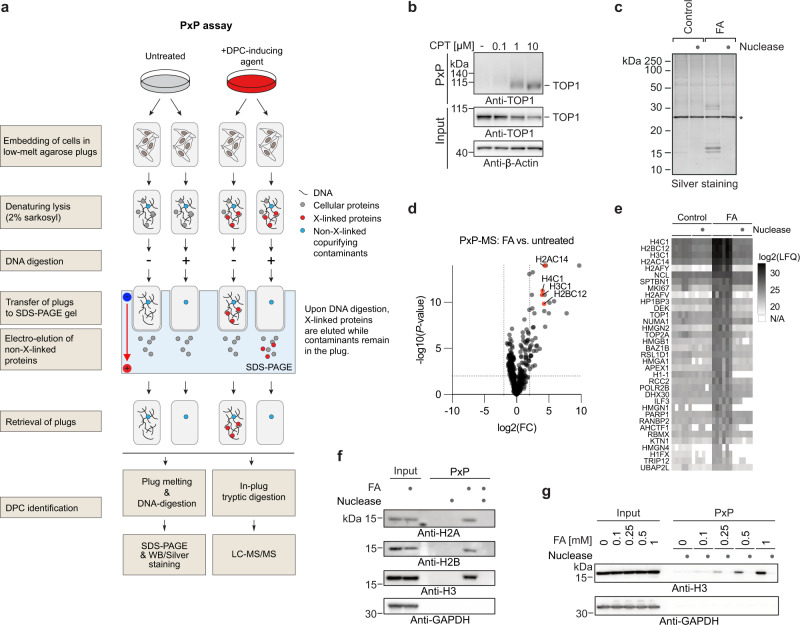
A strategy for the purification of crosslinked proteins. **a** Schematic depiction of the Purification of x-linked Proteins (PxP) assay. Cells are harvested and embedded in low-melt agarose plugs. Plugs are transferred to denaturing lysis buffer. Upon completion of lysis, DNA is optionally digested using a nuclease. Next, plugs are transferred to an SDS-PAGE gel and subjected to electro-elution. For DPC detection, plugs are melted following electro-elution, digested with nuclease and analysed using SDS-PAGE followed by western blotting or silver-staining. Alternatively, plugs are fixed and subjected to in-plug tryptic digestion for quantitative proteomics. **b** Camptothecin (CPT)-induced TOP1-DPC formation assessed by PxP. HeLa T-REx Flp-In cells were treated for 30 min with the indicated doses of CPT prior to isolation of DPCs using PxP and analysis by western blotting. **c** Untreated or formaldehyde (FA)-treated (2 mM, 1 h) HeLa cells were processed as depicted in (**a**) and analysed by SDS-PAGE and silver staining. Asterisk indicates Benzonase nuclease used to digest all samples prior to running the final SDS-PAGE. **d** Mass spectrometry analysis of PxP samples comparing untreated and FA-treated (2 mM, 1 h) HeLa cells. Six plugs per condition were subjected to in-plug tryptic digestion followed by label-free quantitative mass spectrometry. Volcano plot depicting fold change (FC, log2) between conditions plotted against FDR-adjusted *P*-value (-log10). See also Supplementary Data 1, Supplementary Data 2. **e** Heatmap showing normalized intensities of six replicates of statistically significant FA-induced DPCs (FDR-adjusted *P* < 0.01, FC > 2) identified in (**d**) ranked by average intensity upon FA-treatment. See also Supplementary Data 1, Supplementary Data 2. **f** PxP analysis of FA-induced histone crosslinks. Cells were treated for 1 h with 2 mM FA and subjected to PxP extraction including a nuclease digestion as indicated and analysed by western blotting. The experiment was repeated twice and similar results were obtained. **g** PxP analysis of histone H3 crosslinks induced by increasing concentrations of FA. Cells were treated for 1 h with the indicated doses of FA and subjected to PxP analysis including a nuclease digestion as indicated and analysed by western blotting. The experiment was repeated three times and similar results were obtained. Source data are provided as a Source Data file.

The technique described here was inspired by chromosome entrapment experiments that had been designed to investigate interactions between prokaryotic condensin and DNA in *Bacillus subtilis*^34^. In these experiments, bacterial chromosomes were immobilized in low-melt agarose plugs to assess topological interactions with covalently-closed condensin rings. We hypothesized that a similar principle could be utilized to monitor and identify DPCs in mammalian cells. Based on this idea, we designed an assay for the Purification of x-linked Proteins (PxP) (Fig. 1a). In brief, mammalian cells are harvested and embedded in low-melt agarose plugs. Next, plugs are transferred to denaturing lysis buffer containing 2% sarkosyl. Upon completion of cell lysis, plugs are transferred to wells of an SDS-PAGE gel and subjected to electro-elution. During electrophoresis, cellular proteins exit the plug, while DNA (due to its high molecular weight) and crosslinked proteins remain inside. Plugs are then retrieved, melted, and DNA is digested with a nuclease to release the crosslinked proteins. Finally, crosslinked proteins are analysed using SDS-PAGE coupled with western blotting or silver staining. To test our experimental strategy, we first analysed camptothecin (CPT)-induced TOP1-DPCs which formed in a dose-dependent manner with no background signal detectable in untreated cells (Fig. 1b and Supplementary Fig. 1a). Having established that the PxP procedure allows detection of specific DPCs, we next asked whether it can also reveal the identity of non-enzymatic DPCs induced by chemical crosslinkers. To answer this question, we first introduced a control that allows the distinction between co-purifying contaminants and DPCs. Cells of each experimental condition were cast into two plugs. One plug was digested with a nuclease prior to electro-elution, while the second plug was incubated in buffer only. DPCs are expected to elute from the plug upon DNA digestion, while co-purifying contaminants are not (Fig. 1a). We subjected cells to a 1-h formaldehyde pulse, performed PxP extraction, and analysed samples on silver-stained SDS-PAGE gels. Distinct formaldehyde-induced bands could be detected, which were sensitive to nuclease treatment prior to electro-elution, suggesting that treatment with formaldehyde results in crosslinking of specific proteins (Fig. 1c and Supplementary Fig. 1b). To reveal the identity of formaldehyde-induced DPCs, we combined PxP with label-free quantitative proteomics. Plugs were retrieved after electro-elution, fixed, and subjected to in-plug tryptic digestion and detection by LC-MS/MS. Thirty-five proteins were significantly enriched in PxP plugs after formaldehyde exposure (Fig. 1d, e). The most abundant formaldehyde-induced DPCs were formed by core histones (Fig. 1d, e). Histone crosslinking was confirmed by western blotting and could be observed at low formaldehyde concentrations, which did not affect long-term viability (Fig. 1f, g and Supplementary Fig. 1c). We conclude that PxP enables identification of unknown DPCs. In addition, we find that the challenge to preserve genome integrity after formaldehyde exposure is less complex than previously anticipated as formaldehyde-induced DPCs mainly consist of crosslinked nucleosomes.

### Replication-independent repair of 5-azadC-induced DNMT1-DPCs monitored by PxP

**Fig. 2:**
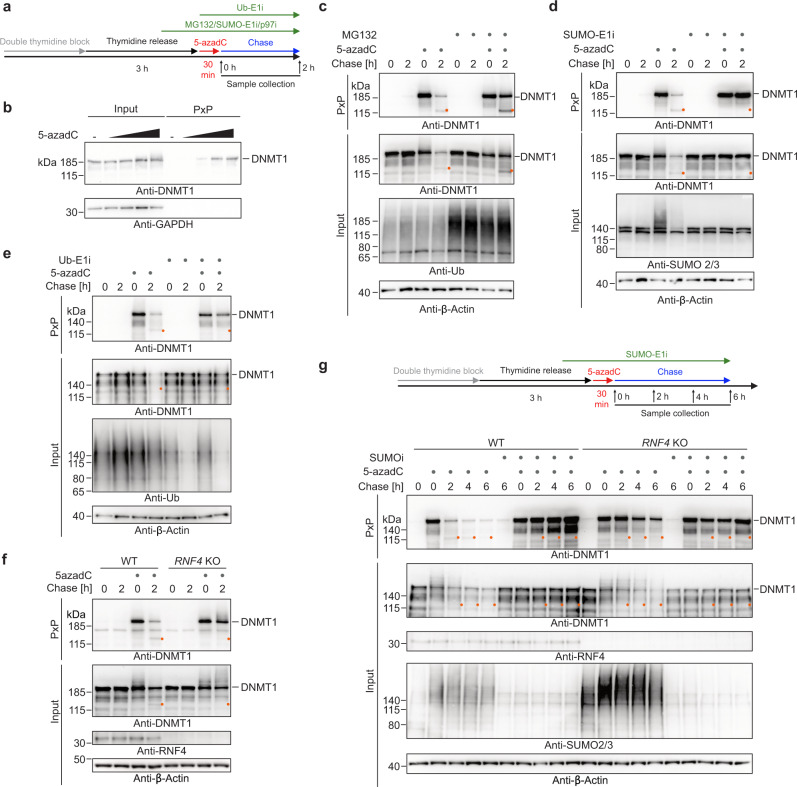
Global-genome repair of 5-azadC-induced DNMT1-DPCs monitored by PxP. **a** Schematic depiction of the experimental workflow used to monitor the repair of 5-azadC-induced DNMT1-DPCs. Cells were synchronized via a double thymidine block and released into early/mid S-phase for 3 h prior to induction of DNMT1-DPCs by a 30-min pulse of 5-azadC. Samples were collected either immediately after 5-azadC exposure or following a chase in drug-free media. Proteasome inhibitor (MG132, 5 µM), p97 inhibitor (p97i CB-5083, 5 µM) and SUMOylation inhibitor (SUMO-E1i ML-792, 5 µM) were added 1 h prior to induction of DPCs and kept during the chase with 5-azadC-free medium. Ubiquitylation inhibitor (Ub-E1i TAK-243, 1 µM) was added together with 5-azadC. **b** 5-azadC-induced DNMT1-DPC formation assessed by PxP. HeLa T-REx Flp-In cells were treated as depicted in (**a**) with the indicated doses of 5-azadC for 30 min prior to immediate isolation of DPCs using PxP and western blotting analysis. **c**–**f** 5-azadC-induced DNMT1-DPC formation and repair upon proteasome inhibition (**c**), inhibition of SUMOylation (**d**), inhibition of ubiquitylation (**e**), or knock-out of *RNF4* (**f**) assessed by PxP. HeLa T-REx Flp-In cells were treated as depicted in (**a**) prior to extraction of DPCs using PxP, and analysis of samples by western blotting using the indicated antibodies. **g** HeLa WT and *RNF4* knock-out (KO) cells were treated and analysed as depicted including an optional treatment with SUMOylation inhibitor (SUMO-E1i ML-792, 5 µM), prior to extraction of DPCs using PxP and analysis of samples by western blotting using the indicated antibodies. Experiments in (**c**–**g)** were repeated three times and similar results were obtained. Source data are provided as a Source Data file.

5-azadC-induced DNMT1-DPCs form post-replicatively and are, thus, an ideal model lesion to study replication-independent DPC repair^25^. Therefore, we tested whether PxP can be used to track the fate of DNMT1-DPCs. We synchronized cells using a double thymidine block, released them into early/mid S-phase, and subjected them to a 30-minute pulse of increasing 5-azadC concentrations (Fig. 2a). Using PxP followed by western blotting, we observed dose-dependent formation of DNMT1-DPCs (Fig. 2b), which were sensitive to nuclease treatment prior to electro-elution (Supplementary Fig. 2a, b). To monitor repair of DNMT1-DPCs, we harvested cells either immediately after 5-azadC treatment or following a chase in drug-free media for 2 h (Fig. 2a). The bulk of DNMT1-DPCs was repaired during the chase, which, in agreement with a previous report^25^, was blocked by pre-treating cells with proteasome inhibitor MG132 (Fig. 2c), by depleting the sole SUMO E2 conjugating enzyme UBC9 (Supplementary Fig. 2c), and by chemical inhibition of SUMO-E1 or ubiquitin-E1 activating enzymes (Fig. 2d, e). Because pre-treatment with ubiquitin-E1 inhibitor interfered with DNMT1-DPC formation (Supplementary Fig. 2d), it was added together with 5-azadC (see scheme in Fig. 2a). Moreover, chemical inhibition of the ATPase p97, which is required for proteasomal degradation of many chromatin proteins^35^, blocked bulk DNMT1-DPC repair (Supplementary Fig. 2e). Interestingly, we noted the appearance of a faster migrating DNMT1-DPC species in PxP and input samples 2 h after 5-azadC exposure (Fig. 2c–e and Supplementary Fig. 2c–e, orange dots). While this species increased upon proteasome or p97 inhibition (Fig. 2c and Supplementary Fig. 2e, orange dots), it was absent after blocking of either SUMOylation or ubiquitylation (Fig. 2d, e and Supplementary Fig. 2c, orange dots). Taken together, this indicated to us that DNMT1-DPCs are proteolytically cleaved in a SUMO- and ubiquitin-dependent manner by an alternative DPC protease, which occurs in parallel to the previously reported proteasomal degradation.

DPC SUMOylation and subsequent ubiquitylation have been proposed to rely on the SUMO E3 ligase PIAS4 and the SUMO-targeted ubiquitin ligase RNF4^24,25^. However, we did not observe a reduction in DPC degradation upon siRNA-mediated depletion or knock-out of *PIAS4* (Supplementary Fig. 2f, g), perhaps indicating redundancy with another SUMO-E3 ligase. In contrast, knock-out of *RNF4* resulted in clear reduction of bulk degradation and reduced formation of the putative DNMT1-DPC cleavage fragment (Fig. 2f). Consistently and in line with a previous report^25^, we found *RNF4* knock-out (KO) cells to be sensitive to 5-azadC exposure (Supplementary Fig. 3a, b). Of note, while RNF4 depletion clearly delayed repair, we observed residual degradation and appearance of the cleaved DNMT1 fragment after a prolonged chase period of up to 6 h (Fig. 2g). Residual repair in *RNF4* KO cells was blocked by chemical inhibition of SUMO- or ubiquitin E1-activating enzymes (Fig. 2g and Supplementary Fig. 3c), suggesting that a second SUMO-targeted ubiquitin ligase activity acts as a, albeit less efficient, back-up to RNF4. We conclude that in addition to the proteasome a second proteolytic activity acts downstream of SUMO-targeted ubiquitylation during global-genome DPC repair.

### SPRTN cleaves DNMT1-DPCs

**Fig. 3:**
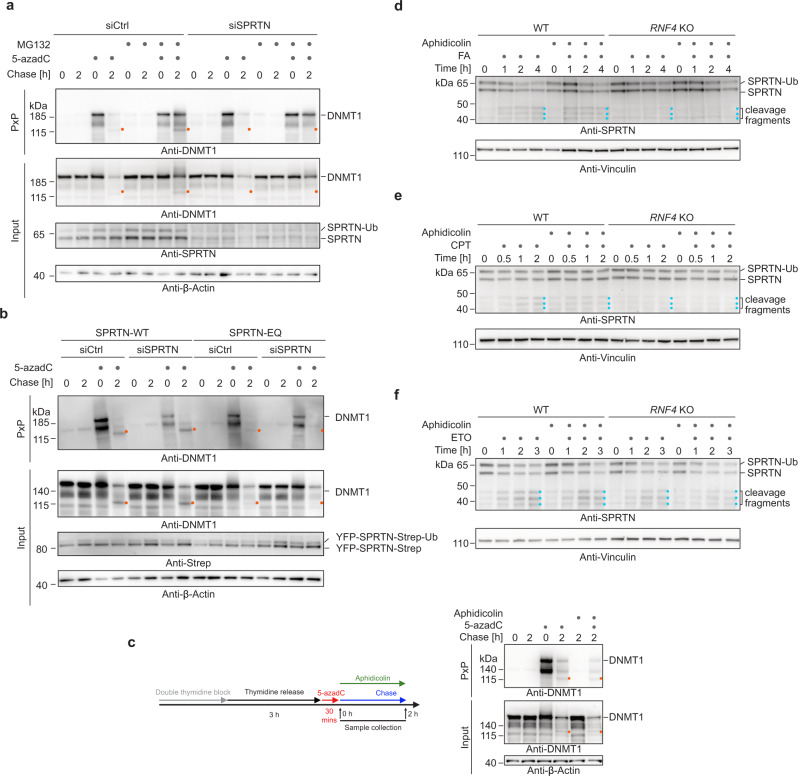
The metalloprotease SPRTN cleaves DNMT1-DPCs during global-genome repair. **a** HeLa T-REx Flp-In cells transfected with the indicated siRNAs were treated as depicted in Fig. 2a. DNMT1-DPCs were isolated using PxP and analysed by western blotting using the indicated antibodies. **b** HeLa T-REx Flp-In cells stably expressing siRNA-resistant SPRTN variants (wildtype (WT) or catalytically inactive E112Q (EQ)) were transfected with the indicated siRNAs and treated as in Fig. 2a. DPCs were isolated by PxP and analysed by western blotting using the indicated antibodies. **c** 5-azadC-induced DNMT1-DPC repair upon inhibition of DNA synthesis assessed by PxP. HeLa T-REx Flp-In cells were treated as depicted including an optional addition of aphidicolin (3 µM) during the chase (left). DNMT1-DPCs were isolated using PxP and analysed by western blotting using the indicated antibodies (right). **d**–**f** HeLa WT or *RNF4* KO cells were treated with formaldehyde (FA, 250 µM) (**d**), camptothecin (CPT, 500 nM) (**e**) or etoposide (ETO, 50 µM) (**f**), including a 2-h pre-treatment with aphidicolin, as indicated, before whole cell lysates were analysed by western blotting using the indicated antibodies. Source data are provided as a Source Data file.

The DPC-specific metalloprotease SPRTN is currently believed to act exclusively at the replication fork^14,17,20,36^. Surprisingly however, siRNA-mediated depletion of SPRTN completely abolished the appearance of the faster-migrating DNMT1-DPC species even upon proteasome inhibition, while neither bulk degradation nor DPC formation were affected (Fig. 3a, orange dots). The appearance of the DNMT1-DPC fragment was restored by expression of a siRNA-resistant version of SPRTN-WT but not by catalytically-inactive SPRTN-E112Q (EQ) (Fig. 3b, orange dots). These data suggest that the observed DNMT1-DPC fragment is a product of SPRTN-dependent proteolysis. DNMT1-DPCs form in the wake of DNA synthesis, therefore it seemed unlikely that the cleaved DPC is a consequence of SPRTN’s established role in replication-coupled DPC repair. Indeed, inhibition of DNA synthesis by aphidicolin following induction of DNMT1-DPCs had no effect on SPRTN-dependent DPC cleavage or bulk repair (Fig. 3c, orange dots, and Supplementary Fig. 4a). Moreover, cleavage was not affected by knock-out of the adaptor protein TEX264 (Supplementary Fig. 4b, orange dots), which was shown previously to be involved in replication-coupled repair of DPCs by SPRTN^36^. We also excluded an involvement of transcription, because inhibition of RNA synthesis using the CDK9-inhibitor flavopiridol showed no effect on DPC cleavage or repair (Supplementary Fig. 4c,d, orange dots).

To test whether SPRTN also responds to other types of DPCs in a replication-independent manner, we monitored autocleavage of the protease, an indicator of SPRTN activation^13,14,37^. We treated cells with formaldehyde (thereby inducing histone-DPCs), CPT (TOP1-DPCs), or etoposide (ETO, causing TOP2-DPCs) and monitored accumulation of SPRTN autocleavage fragments over time. Formaldehyde- and CPT-induced autocleavage was strongly reduced in *RNF4* KO cells, suggesting that SPRTN activation by TOP1- and histone-DPCs occurs similar to what we observed upon post-replicative induction of DNMT1-DPCs (Fig. 3d, e, blue dots). Interestingly, etoposide-induced SPRTN autocleavage occurred largely independent of RNF4 and was partially reduced by aphidicolin in *RNF4* KO cells (Fig. 3f, blue dots), indicating that TOP2-DPCs are sensed and signalled differently. In the case of CPT and FA however, SPRTN autocleavage was completely unaffected by inhibition of DNA synthesis using aphidicolin (Fig. 3d, e, blue dots, and Supplementary Fig. 4e). This was in contrast to SPRTN’s role at replication forks, which relies on DNA polymerases extending nascent strands up to the protein adduct^20^. We thus conclude that SPRTN responds to various DPCs in a global-genome manner that does not rely on the replication machinery to detect the lesion. Next, we asked whether global-genome DPC repair by SPRTN is also active outside the S/G2-phase (when SPRTN expression levels are high^38^). We arrested cells using the CDK4/CDK6 inhibitor palbociclib in early G1 phase (Supplementary Fig. 4f), which was accompanied by a strong reduction in SPRTN protein levels (Supplementary Fig. 4g–i). Low levels of SPRTN expression made the assessment of autocleavage impossible, but also indicated that it is unlikely that the protease is important in G1 phase. Collectively, these data demonstrate that SPRTN targets DPCs during global-genome repair downstream of SUMO-targeted ubiquitylation and that this mechanism, while being replication-independent, primarily operates in the S/G2 phase of the cell cycle.

### SPRTN patient variants affect replication-independent DNA-protein crosslink repair

**Fig. 4:**
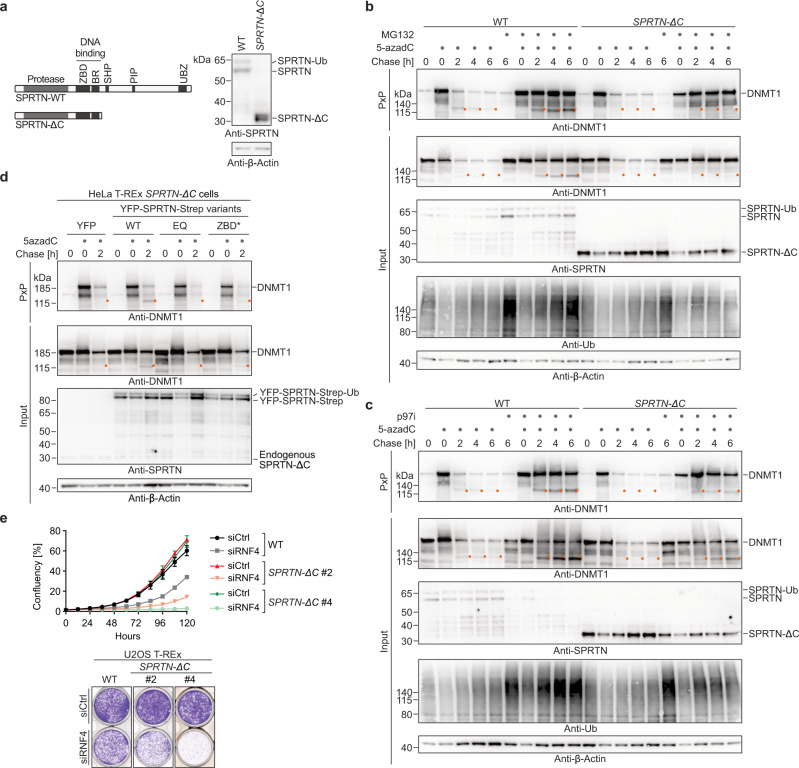
SPRTN patient variants affect global-genome DNA-protein crosslink repair. **a** Domain structure of SPRTN wildtype and the C-terminally truncated SPRTN-ΔC variant observed in Ruijs-Aalfs syndrome patients indicating SPRTN’s two DNA binding domains (zinc-binding domain, ZBD, and basic region, BR) and interaction motifs/domains for binding to p97 (SHP), PCNA (PIP) and ubiquitin (UBZ) (left). Western blot analysis of HeLa T-REx Flp-In cells genetically-engineered to express patient-like SPRTN-ΔC variants (right). **b**, **c** 5-azadC-induced DNMT1-DPC repair assessed by PxP in WT and *SPRTN-ΔC* cells. HeLa T-REx Flp-In cells were treated as depicted in Fig. 2g including a pre-treatment with proteasome inhibitor MG132 (**b**) or inhibition of p97 (p97i) (**c**), as indicated. Upon isolation of DPCs by PxP, samples were analysed using western blotting using the indicated antibodies. **d** HeLa T-REx Flp-In *SPRTN-ΔC* cells were complemented with YFP, YFP-SPRTN-Strep variants as indicated (wildtype (WT), E112Q (EQ), R185A (ZBD*)) and treated as shown in Fig. 2a, before DPCs were isolated using PxP and analysed by western blotting using the indicated antibodies. **e** U2OS T-REx Flp-In WT and *SPRTN-ΔC* cells were transfected with the indicated siRNAs. Cell confluency was monitored over 5-days using IncuCyte live cell imaging, (top, values represent the mean ± SD of 3 technical replicates), before cells were stained with crystal violet (bottom). Source data are provided as a Source Data file.

Ruijs-Aalfs syndrome is caused by partial loss-of-function *SPRTN* mutations and is characterized by progeroid features and early onset hepatocellular carcinomas^18,39^. Intriguingly, several aspects of the disease are difficult to reconcile with a purely replicative function of SPRTN. Patients and mice bearing hypomorphic *SPRTN* mutations display signs of failed tissue homeostasis in the largely quiescent liver and in postmitotic lens epithelial cells^18,19,39^. In contrast, the highly proliferative haematopoietic system, which is in addition challenged by high endogenous formaldehyde concentrations^40^, seems not to be affected. Therefore, we asked whether replication-independent cleavage of DPCs may be affected by patient variants. To investigate this question, we engineered cells to express patient-mimicking variants. We edited the endogenous *SPRTN* locus in HeLa T-REx Flp-In cells using two gRNAs resulting in the deletion of the entire coding region of exon 5 (Supplementary Fig. 5a). The resulting mutant cells express a SPRTN-ΔC variant, which is highly reminiscent of the truncated SPRTN variants observed in Ruijs-Aalfs syndrome patients (Fig. 4a and Supplementary Fig. 5a). While *SPRTN-ΔC* cells were viable, they failed to efficiently cleave DNMT1-DPCs; the DPC cleavage band observed during a 6-h chase in WT cells was hardly detectable in mutant cells (Fig. 4b, c). Residual amounts of DPC cleavage fragments were only observed upon inhibition of proteasomal degradation or p97 activity (Fig. 4b, c). Re-expression of SPRTN-WT, but not of SPRTN-EQ rescued the cleavage of DNMT1-DPCs in *SPRTN-ΔC* cells (Fig. 4d). A catalytically-compromised DNA-binding mutant SPRTN-ZBD* (R185A)^23,41^ displayed strongly reduced activity (Fig. 4d). Despite being unable to efficiently cleave DNMT1-DPCs, *SPRTN-ΔC* cells were not sensitive to exposure of 5-azadC (Supplementary Fig. 5b), likely due to redundant DPC degradation by the proteasome. In line with SPRTN acting downstream of RNF4, 5-azadC sensitivity caused by depletion of RNF4 was comparable in *SPRTN-ΔC* and in wild-type HeLa T-REx Flp-In cells (Supplementary Fig. 5b). In addition, we noted that RNF4 depletion resulted in mild synthetic growth defects in *SPRTN-ΔC* cells (Supplementary Fig. 5c), indicating a complex relationship between both factors (see Discussion). We also observed that siRNA-mediated depletion of SPRTN resulted in growth defects in *RNF4* KO cells and led to increased 5-azadC sensitivity (Supplementary Fig. 5d, e). To corroborate these results, we generated U2OS T-REx Flp-In *SPRTN-ΔC* cells by generating frameshift mutations using a single gRNA, which targets the beginning of exon 5 (Supplementary Fig. 5f). In U2OS *SPRTN-ΔC* cells, DNMT1-DPC cleavage was defective (Supplementary Fig. 5g), and depletion of RNF4 caused synthetic lethality/viability defects (Fig. 4e and Supplementary Fig. 5h). Taken together, these results show that SPRTN patient variants compromise replication-independent DPC repair. Moreover, our data indicate that cells can tolerate such reduced repair capacity in principle, but only if proteasomal DPC repair is fully functional.

### Compromised ubiquitin binding is the main defect of SPRTN patient variants

**Fig. 5:**
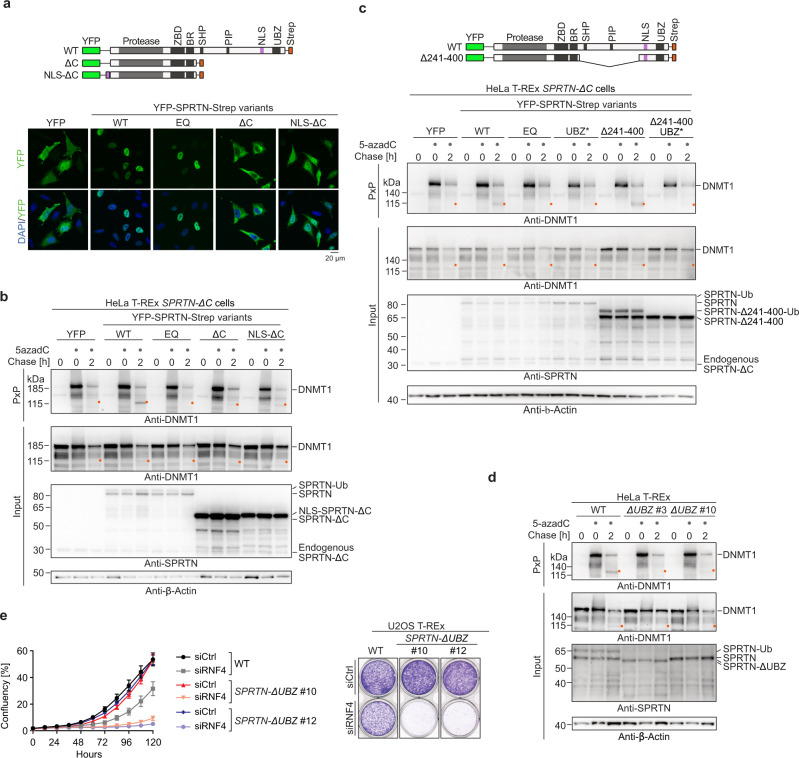
Compromised ubiquitin binding is the main defect of SPRTN patient variants. **a** Schematic depiction of YFP-SPRTN-Strep variants used to complement HeLa T-REx Flp-In *SPRTN-ΔC* cells (top). The localisation of each variant (wildtype (WT), E112Q (EQ), ΔC, and ΔC including an orthogonal N-terminal nuclear localisation signal (NLS-ΔC)) was determined using immunofluorescence staining with anti-GFP antibodies recognizing YFP. Please note that different exposure times are shown for different variants due to different expression levels, see western blot analysis in (**b**). **b** HeLa T-REx Flp-In *SPRTN-ΔC* cells complemented with the indicated SPRTN variants were treated as depicted in Fig. 2a, DPCs were isolated using PxP, and samples were analysed by western blotting using the indicated antibodies. **c** HeLa T-REx Flp-In *SPRTN-ΔC* cells complemented with indicated SPRTN variants (full length or an internally-truncated version lacking amino acids 241–400 (Δ241–400) either WT or in combination with an amino acid replacement within the UBZ domain (D473A, UBZ*), were treated as depicted in Fig. 2a. DPCs were isolated using PxP and analysed by western blotting using the indicated antibodies. **d** Two clonal HeLa T-REx Flp-In *SPRTN-ΔUBZ* cell lines (see Supplementary Fig. 7a) were treated as depicted in Fig. 2a, before isolation of DPCs and analysis by western blotting using the indicated antibodies. **e** U2OS T-REx Flp-In WT and *SPRTN-ΔUBZ* cells were transfected with the indicated siRNAs. Cell confluency was monitored over 5-days using IncuCyte live cell imaging, (left, values represent the mean ± SD of 3 technical replicates), before cells were stained with crystal violet (right). Experiments in (**a)** and (**c)** were performed three times and experiments in (**b)** and **(d)** twice with similar results. Source data are provided as a Source Data file.

Next, we asked why patient variants fail to efficiently cleave DNMT1-DPCs. It has previously been speculated that the major defect of SPRTN-ΔC variants is their mislocalisation to the cytosol due to loss of a C-terminal nuclear localisation signal (NLS)^15,18^. Therefore, we complemented HeLa T-REx Flp-In *SPRTN-ΔC* cells with YFP-tagged SPRTN-ΔC constructs either carrying an additional N-terminal NLS or not (Fig. 5a, top). As expected, SPRTN-ΔC was mislocalised to the cytosol, while NLS-SPRTN-ΔC was found preferentially in the nucleus (Fig. 5a, bottom). Nevertheless, NLS-SPRTN-ΔC was not able to fully restore DNMT1-DPC cleavage in *SPRTN-ΔC* cells (Fig. 5b). Both ΔC variants showed only a slight increase in DPC cleavage, despite being heavily overexpressed and present at much higher levels than SPRTN-WT, which efficiently rescued cleavage (Fig. 5b). Taken together, these results suggest that mislocalisation is not the sole defect of SPRTN-ΔC variants and that the C-terminal part of SPRTN contains an additional critical feature required for replication-independent DPC cleavage. In addition to ensuring nuclear localisation, SPRTN’s C-terminal tail contains three protein-protein interaction motifs: a SHP-box (SHP) mediating binding to p97^38,42^, a PIP-box (PIP) for interacting with PCNA^43^, and a ubiquitin-binding zinc finger (UBZ)^43^. In order to identify the critical domain for DPC cleavage, we complemented *SPRTN-ΔC* cells with SPRTN variants bearing replacements of key amino acids in all three motifs. Expression of SPRTN-WT or PIP*- and SHP*-mutant variants restored DPC cleavage, while SPRTN variants with a defective UBZ domain (D473A - UBZ*) appeared to display reduced cleavage (Supplementary Fig. 6a). To further corroborate that PIP- and SHP-box are dispensable, we complemented *SPRTN-ΔC* cells with a SPRTN variant lacking the entire region between SPRTN’s DNA binding domains and the C-terminal NLS and UBZ domain (Fig. 5c, top). Despite lacking both, PIP- and SHP-box, this variant (SPRTN-Δ241-400) fully supported DNMT1-DPC cleavage, unless its UBZ domain was defective as well (SPRTN-Δ241-400-UBZ*) (Fig. 5c, bottom). We conclude that PCNA and p97 binding domains are not required for SPRTN’s function in replication-independent DPC repair, while ubiquitin binding appears to be crucial. In addition to recruiting SPRTN to sites of DNA damage, the UBZ domain is also required for stabilising monoubiquitylation of SPRTN (Supplementary Fig. 6a)^38,42,43^, which in turn regulates SPRTN autocleavage^37^. To exclude that the loss of monoubiquitylation is causative for the DNMT1-DPC cleavage defects of SPRTN-UBZ*, we tested a linear fusion of ubiquitin to SPRTN-UBZ* (SPRTN-UBZ*-Ub), which we showed previously to restore the regulation of SPRTN autocleavage^37^. However, we observed that this variant remained unable to cleave DNMT1-DPCs (Supplementary Fig. 6b). To further exclude that the reduction of DPC cleavage by SPRTN-UBZ* is a consequence of reduced catalytic activity, we assessed the activity of the recombinant enzyme in vitro, using cleavage of a DPC model substrate (Protein G-oligonucleotide conjugates^23,44^) and autocleavage as a readout. While SPRTN-ΔC showed slightly reduced substrate cleavage and autocleavage, SPRTN-UBZ*’s activity was indistinguishable from the WT enzyme (Supplementary Fig. 6c,d).

Next, we wanted to extend our observations to endogenously expressed SPRTN. We edited the endogenous locus using a gRNA that targets the coding sequence of SPRTN’s C-terminal UBZ domain to generate *SPRTN-ΔUBZ* variants. We obtained one *SPRTN-ΔUBZ* clone (#3) with homozygous deletions resulting in premature stop codons. As a consequence, key residues of the UBZ domain are lost (Supplementary Fig. 7a). DNMT1-DPC cleavage was virtually absent in *SPRTN-ΔUBZ* clone #3, confirming that the UBZ domain is critically required for SPRTN’s global-genome repair function (Fig. 5d). Sequencing analysis of a second clone (#10) revealed that one allele contained an in-frame deletion resulting in the loss of key UBZ features (#10 Allele 2, Supplementary Fig. 7a). The second allele of clone #10 was identified to bear a premature stop codon, however only downstream of all important UBZ residues (#10 Allele 1, Supplementary Fig. 7a). Clone #10 displayed residual SPRTN monoubiquitylation (Fig. 5d), which is consistent with residual UBZ function. In agreement with clone #10 retaining residual levels of ubiquitin binding, we observed minor degrees of DNMT1-DPC cleavage (Fig. 5d). A key role for the UBZ domain was further indicated by CPT-, formaldehyde-, and ETO-induced autocleavage being virtually absent in *SPRTN-ΔUBZ* #3 cells (Supplementary Fig. 7b–d). As observed in *SPRTN-ΔC* cells, RNF4 depletion caused growth defects in HeLa T-REx *SPRTN-ΔUBZ* cells (Supplementary Fig. 7e, f), while resulting in synthetic lethality in U2OS T-REx *SPRTN-ΔUBZ* cells (Fig. 5e, Supplementary Fig. 7g. We conclude that *SPRTN-ΔUBZ* fully phenocopies the effect of Ruijs-Aalfs syndrome patient variants, suggesting that loss of ubiquitin-binding is the key defect of SPRTN-ΔC.

## Discussion

DNA lesions are diverse in nature and are studied using a broad variety of lesion-specific techniques. DPCs have only recently emerged as important endogenous lesions, and the available toolkit to investigate these adducts is therefore limited. We developed a DPC extraction method that is compatible with various downstream readouts and is able to detect and identify DPCs in various experimental scenarios. We have combined PxP with quantitative proteomics to reveal that formaldehyde induces less complex DPCs than anticipated. Since formaldehyde is a major source of endogenous DNA damage^8,40^, our data indicate that nucleosomal histone-DNA crosslinks are frequent genotoxic challenges faced by mammalian cells.

**Fig. 6:**
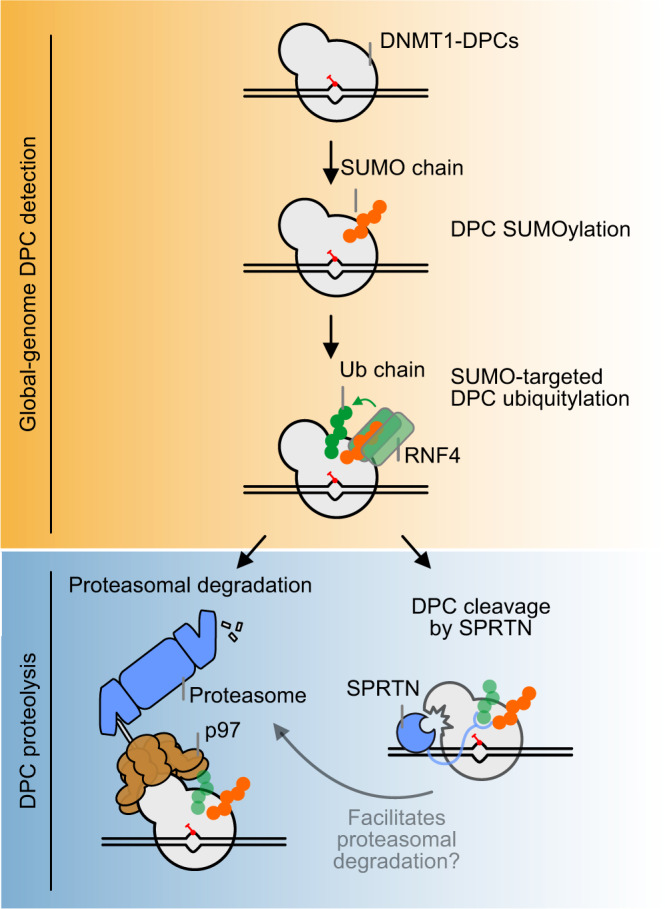
Model of global-genome DNMT1-DPCs repair. Repair of 5-azadC-induced DNMT1-DPCs is initiated by SUMOylation, followed by subsequent ubiquitylation by the SUMO-targeted ubiquitin ligase RNF4. Modified DNMT1-DPCs are either targeted by p97- and proteasome-dependent degradation or cleaved by SPRTN. The DPC fragment generated by SPRTN cleavage may be subjected to further degradation by p97 and the proteasome.

By studying the repair of 5-azadC-induced DNMT1-DPCs with PxP, we discovered an unexpected role of the SPRTN metalloprotease in replication-independent DPC repair (Fig. 6). Intriguingly, replication-independent DPC cleavage by SPRTN relies on the same initial signals as proteasomal degradation^24,25^, namely SUMO-targeted ubiquitylation by RNF4. DPC detection by the SUMO system appears to occur in a global-genome manner that does not rely on transcription or replication machineries to detect the lesion. Despite being replication-independent, SPRTN-mediated global-genome repair seems restricted to the S/G2 phase, due to low SPRTN expression in G1 cells. The fact that SPRTN-dependent cleavage increases upon proteasome inhibition, suggests that SPRTN acts independently of proteasomal degradation. In addition, we observed that inhibition of the ATPase p97 inhibits proteasomal DPC degradation, while increasing the abundance of the SPRTN-dependent DPC cleavage fragment. p97 has the ability to unfold substrate proteins by threading them through its central pore, which often results in their degradation^35^. Therefore, we propose that SUMO-targeted ubiquitylation results in (a) p97-dependent extraction and subsequent proteasomal degradation or (b) SPRTN-dependent cleavage, perhaps if extraction is inefficient. However, the fate of the DPC fragment produced by SPRTN cleavage remains unclear. It is possible that the cleavage fragment accumulates upon proteasomal inhibition (while it appears only transiently, if proteasome is active), because proteasome and SPRTN are two independent parallel mechanisms that target DNMT1-DPCs. Alternatively, the accumulation of the DNMT1-DPC cleavage fragment upon proteasomal inhibition may indicate that it is itself a substrate for proteasomal degradation. In this hypothetic model, SPRTN cleavage may facilitate proteasomal degradation of DPCs by generating a novel N-terminus, which could trigger additional DPC ubiquitylation by N-end rule E3 ubiquitin ligases. We favour the second scenario because it seems unlikely that the generation of the 115 kDa DNMT-DPC fragment is in itself sufficient for repair.

The fact that SPRTN patient variants displayed compromised global-genome DPC repair may explain the defects observed in non-replicative tissues of Ruijs-Aalfs syndrome patients. PxP in combination with patient-mimicking *SPRTN-ΔC* cells enabled us to conduct a detailed structure-function analysis of critical features within SPRTN for global-genome DPC cleavage. Notably, DPC cleavage was strongly reduced upon mutation of SPRTN’s single-stranded DNA binding domain, the ZBD. This observation suggests that DNMT1-DPCs contain a DNA structure with single-stranded or unpaired DNA features, which were shown in vitro to be required for SPRTN activity (2-3 unpaired bases suffice for activation^23^). Interestingly, within the DNMT1-DPC, the 5-azadC base is flipped-out of the DNA duplex into the enzyme’s active site, which destabilize the DNA helix locally and result in additional flipped-out bases^45,46^. Structural data indicate that these bases would be accessible for other proteins^46^, which would allow SPRTN binding and, thus, activation. Alternatively, DNMT1-DPCs may require pre-processing by a yet to be identified helicase or nuclease activity prior to SPRTN cleavage. The p97-binding motif of SPRTN had no influence on DPC cleavage, which is in line with our observation that p97 activity is only required for proteasomal degradation. In agreement with SPRTN’s role in DNMT1-DPC repair being replication-independent, binding to the replication clamp PCNA by SPRTN was also not necessary for activity. In addition to losing p97 and PCNA binding, SPRTN-ΔC variants have three further defects; mislocalisation, reduced DPC cleavage activity, and loss of ubiquitin binding. Importantly however, loss of the UBZ domain alone was sufficient to recapitulate the phenotypes of SPRTN-ΔC suggesting that ubiquitin binding is the critical feature lacking in Ruijs-Aalfs syndrome-associated SPRTN variants. One function of the UBZ domain is the establishment of SPRTN monoubiquitylation^38^, which regulates SPRTN autocleavage^37^. However, a linear ubiquitin fusion, which restores autocleavage^37^, did not restore DPC cleavage. Therefore, we favour the interpretation that the primary defect of SPRTN-ΔC and SPRTN-ΔUBZ variants is the inability to recognize RNF4-catalyzed DPC ubiquitylation. In support, RNF4 modifies DPCs primarily with K48-linked ubiquitin chains^25^, which matches the ability of SPRTN’s UBZ to interact with such chains^42^.

Our findings that efficient SPRTN-dependent DNMT1-DPC cleavage and formaldehyde- and CPT-induced SPRTN-autocleavage require the presence of RNF4 suggest an epistatic interaction between the two enzymes. Interestingly, however, the genetic relationship between both DPC repair factors is more complex. While SPRTN acts downstream of RNF4 during global-genome repair (this study), it also functions during replication-coupled repair^14,20^, which is independent of RNF4^25^. Moreover, in both scenarios SPRTN functions in addition to proteasomal DPC degradation (which is RNF4-dependent outside of replication^24,25^). In agreement, *RNF4* KO cells were 5-azadC sensitive (because SPRTN and proteasomal repair are affected), while *SPRTN-ΔC* cells were not (because proteasomal global-genome repair is still available). In contrast, siRNA-mediated depletion of SPRTN results in increased 5-azadC sensitivity in *RNF4* KO cells, which may reflect a synthetic defect between a reduction in both global-genome DPC repair branches (SPRTN and proteasome) combined with a reduction of SPRTN’s replication-coupled repair function. Interestingly, in scenarios of impaired SPRTN function (*SPRTN-ΔC, SPRTN-ΔUBZ*), additional depletion of RNF4 resulted in synthetic viability defects, which are presumably caused by unrepaired endogenous DPCs. Some DPCs may rely more on SPRTN-dependent cleavage than proteasomal degradation, perhaps explaining the differences between 5-azadC sensitivity and viability. Notably, in the absence of RNF4 or upon SPRTN mutation, global-genome cleavage of DPCs was strongly reduced but did still occur. Therefore, the synthetic phenotype may also be caused by simultaneous partial loss-of-function at two critical points of the same pathway.

To conclude, DPCs are not only repaired by replication-coupled mechanisms but are also efficiently targeted by SUMO-dependent global-genome DPC repair mechanisms, that are replication- and transcription-independent. What determines pathway choice during DPC repair and whether it is linked to genomic context is an exciting open question. Furthermore, whether transcription-coupled DPC repair occurs as well remains to be determined. We anticipate that the PxP methodology will be instrumental to address these key questions on DPC repair.

## Methods

### Cell lines

HeLa, U2OS T-REx Flp-In and HeLa T-REx Flp-In cells were provided by Cell Services, The Francis Crick Institute, and grown in Dulbecco’s Modified Eagle Medium (DMEM) supplemented with 10% (v/v) fetal bovine serum (FBS). HeLa T-REx Flp-In cells stably expressing siRNA-resistant YFP-SPRTN-Strep-tag and HeLa T-REx Flp-In *SPRTN-ΔC* cells expressing SPRTN variants were generated using the Flp-In system (pOG44, V600520, Thermo Scientific) according to manufacturer’s instructions and selected in Hygromycin B (150 µg/ml) (10687010, Thermo Fisher). Protein expression was induced by overnight incubation with doxycycline (D9891, Sigma) (final concentration 1 µg/ml).

### Generation of cell lines

Genome-edited cell lines were generated by transfection of pX330 plasmids (#82580, Addgene) encoding the following gRNA sequences: HeLa *RNF4* KO cells (gRNA_RNF4#1 GCTACTCAGAGAAAGCGTCG); U2OS T-REx Flp-In *PIAS4* KO cells (gRNA_PIAS4#1 AGCACGGGGTAGTCAATAT); U2OS T-REx Flp-In *SPRTN-ΔC* cells (gRNA_SPRTN-ΔC#3 ACTAAAAGGGATTACTAGCT); HeLa T-REx Flp-In and U2OS T-REx Flp-In *SPRTN-ΔUBZ* cells (gRNA_SPRTN-ΔUBZ#1 CACTTGGACTGGTGCCTTGA). HeLa T-REx Flp-In *SPRTN-ΔC* cells were generated by co-transfection of two pX330 plasmids containing two different gRNAs (gRNA_SPRTN-ΔC#1 TTGGCAGATAAACCCAACAG and gRNA_SPRTN-ΔC#2 ATTAACCAGAACTTCCTGAC). 16 h after transfection of plasmids using Lipofectamine 2000 (11668030, Thermo Scientific), cells were selected in puromycin-containing (1 µg/ml) media for 48 (HeLa T-REx Flp-In or HeLa cells) or 72 (U2OS T-REx Flp-In cells) hours. Next, cells were seeded in 96 well plates in a concentration of 0.75 cells per well. Single colonies were transferred once confluency was reached and editing efficiency was confirmed by western blotting and Sanger sequencing. Polyclonal HeLa T-REx Flp-In *TEX264* KO cells were generated using two different gRNAs (gRNA_TEX264#1 ATAAGTGCCGATGTGCCGT and gRNA_TEX264#2 CTGTGTGCCTATCCTCGGC) with a gRNA targeting the safe-harbour-site *AAVS1* (gRNA_AAVS1#1 GTCCCTAGTGGCCCCACTGT) as control. Editing efficiency of polyclonal pools was confirmed by western blotting following selection and cells were directly used for experiments without selecting single clones. All cell lines generated in this study are available from the corresponding author upon request.

### Genotyping of single clones

Genomic DNA of single clones was extracted by lysing cells in 5 mM Tris-HCl pH 8 at 99 °C for 2 min, followed by addition of proteinase K (0.1 mg/ml, 25530049, Invitrogen). Samples were then incubated at 55 °C for 5 h, before proteinase K was heat-inactivated for 45 min at 85 °C. 10 ng of genomic DNA was used as template to amplify the edited region while adding overhangs homologous to the pDONR221 vector (see Supplementary data 3 for primer sequences used for each genotype) using Platinum II Hot-Start Green PCR Master Mix (14001012, Thermo Fischer). Next, PCR products were gel-purified (REF 740611, MACHEREY-NAGEL) and cloned by TEDA-based cloning^47^ into a pDONR221 backbone amplified with Q5® Hot Start High-Fidelity 2X Master Mix (M0494S, NEB). Plasmid DNA was isolated from at least five single colonies and analysed by Sanger sequencing.

### siRNA transfection

For PxP experiments, cells were transfected in 60 mm dishes. 5 µl siRNA (20 µM) and 12.5 µl Lipofectamine RNAiMAX Transfection Reagent (13778075, Thermo Scientific) were each diluted in 400 µl Opti-MEM Medium. Following a 5 min incubation, siRNA and Lipofectamine RNAiMAX Transfection Reagent dilutions were mixed. After an additional 15 min, the transfection mix was added to cells. After 16 h, cells were reseeded into 60 mm dishes, followed by synchronization using a double thymidine block and PxP extraction 72 h after transfection as described below. For viability and 5-azadC sensitivity assays, siRNA transfections were performed in 6-well plates. 3 µl of siRNA were mixed with 100 µl of Opti-MEM Medium and incubated for 5 min. 7.5 µl of Lipofectamine RNAiMAX were mixed with 100 µl of Opti-MEM Medium and incubated for 5 min. Next, both solutions were mixed, incubated for additional 15 min and added to the well containing 800 µl of media. The following siRNAs (Horizon Discovery) were used: siCTRL (Control pool, D-001810-10-20), siRNF4 (SMARTpool, L-006557-00-0005), siUBC9 (SMARTpool, L-004910-00-0005), siSPRTN#1 (CAAGGAACCAGAGAAUUA) and siPIAS4 (SMARTpool, L-006445-00-0005).

### Purification of x-linked proteins (PxP)

DPCs were induced by addition of methanol-free formaldehyde (28906, Fisher Scientific) or camptothecin (CPT, 208925, Sigma) (concentrations indicated in figure legends) to asynchronous cells. For induction of 5-azadC (A3656, Sigma) induced DNMT1-crosslinks, cells were synchronized using a double thymidine block. In brief, cells were seeded in the morning and thymidine-containing media (2 mM, T9250, Sigma) was added after 8 h. The next day, cells were released in thymidine-free medium for 9 h, prior to readdition of thymidine and overnight incubation. Then, cells were released in thymidine-free medium and treated with 5-azadC (10 µM), MG132 (5 µM) (M7449, Sigma), SUMO-E1 inhibitor ML-792 (5 µM) (Axon Medchem, 3109), Ub-E1 inhibitor TAK-243 (1 µM) (AOB87172, Chemietek), aphidicolin (3 µM) (A4487, Sigma) p97i CB-5083 (5 µM) (HY-12861-10mg, Hölzel) or flavopiridol (10 µM) (F3055, Sigma) as indicated in figures.

For PxP, formaldehyde-treated cells were harvested at the respective time points by trypsinisation and counted, while CPT- and 5-azadC-treated cells were scraped in ice-cold PBS (an additional plate per condition was trypsinised and counted to determine the number of cells per plate). For CPT treatments, plates were prechilled on ice for 5 min before scraping to minimize TOP1cc reversal. Next, cells were washed and resuspended in PBS at 2 × 10^4^ cells/µl (cells were optionally pelleted, frozen and stored at −80 °C at this point, apart from CPT-treated cells, which were processed immediately). 10 µl of the cell suspension were directly lysed in 1x NuPAGE LDS sample buffer (NP0007, Thermo Scientific) to serve as input samples. The remaining cell suspension was pre-warmed for 45 s at 45 °C prior to mixing with an equal volume of low melt agarose (2% in PBS, 1613111, Bio-Rad) and immediately cast into plug molds (#1703713, Bio-Rad) with a total volume of ca. 90 µl. Plugs were placed at 4 °C for 5 min, prior to transfer into 1 ml ice-cold lysis buffer (1 x PBS, 0.5 mM EDTA, 2% sarkosyl, cOmplete EDTA-free protease inhibitor cocktail (4693132001, Merck), 0.04 mg/ml Pefabloc SC (11585916001, Merck). Lysis was carried out on a rotating wheel at 4 °C for 4 h. Following lysis, DNA was optionally digested by nuclease. To this end, plugs were transferred to washing buffer (50 mM Tris/HCl pH 8, 0.5 mM MgCl_2_, 0.01% sarkosyl). After 10 min, buffer was replaced by fresh washing buffer only or washing buffer containing benzonase nuclease (0.2 U/µl, 70746, Merck Millipore), followed by incubation in a thermoshaker (500 rpm, 37 °C) for 1 h. For electro-elution, plugs were transferred to the wells of 10-well SDS-PAGE gels (12%, 1.5 mm Novex WedgeWell or BOLT gels, ThermoFisher). Electrophoresis was carried out in 300 ml MOPS buffer at 20 mA per gel for 60 min in a Mini Gel Tank (ThermoFisher). Following electro-elution, plugs were retrieved and transferred to tubes containing 1 ml washing buffer, while the gel was stained using InstantBlue (ISB1L, Sigma) to confirm successful extraction of non-crosslinked cellular proteins. Plugs were incubated on a rotating wheel at 4 °C for 10 min. Plugs of the same conditions were pooled at this stage of the purification (typically two plugs were cast per condition for CPT-, formaldehyde- and 5-azadC-induced DPCs). The supernatant was aspirated and plugs were melted at 99 °C for 5 min, followed by addition of 20 µl washing buffer containing 50 units benzonase nuclease per plug and incubation at 37 °C for 30 min. Samples were then frozen at −80 °C. For analysis by western blotting, NuPAGE LDS sample buffer was added and samples were subjected to western blotting using the indicated antibodies. For silver staining, frozen samples were thawed and centrifuged in a table-top centrifuge at top speed at 4 °C. Supernatant was then passed through 0.45 µM SpinX centrifuge tube filters (CLS8162, Merck) to remove residual agarose. NuPAGE LDS sample buffer was added and samples analysed using SDS-PAGE in a Bolt 12 % 1.5 mm 10-well gel followed by silver staining (SilverQuest Silver Staining Kit, LC6070, ThermoFisher). For analysis by mass spectrometry, plugs were washed twice following electro-elution in washing buffer and fixed in 40% ethanol/10% acetic acid on a rotating wheel at 4 °C for 1 h. Finally, plugs were washed twice in 100 mM ammonium bicarbonate.

### Western blotting

Samples were boiled in NuPAGE LDS sample buffer (NP0007, Thermo Scientific) containing NuPAGE Sample Reducing Agent (NP0009, Thermo Scientific), before SDS-PAGE using NuPAGE 4–12% 20 well gels (a 4–12% 12- well Bolt gel was used for Fig. 2b). Following electrophoresis, proteins were transferred on 0.45 µm PVDF membranes (IPVH00010, Merck) using a wet transfer system (#1704070, Bio-Rad) for 70 min at 100 V. Membranes were blocked in 5 % milk in TBS-T for 1 h before addition of primary antibody: Anti-DNMT1 (D63A6) antibody (1:1000) (#5032, Cell Signaling), Anti-Actin antibody (1:1000) (Sc-47778, Santa Cruz Biotechnology), Anti-SUMO2/3 antibody (1:2000) (ab3742, Abcam), Anti-TOP1 antibody (1:1000) (ab109374, Abcam), Anti-Ub antibody (1:1000) (Sc-8017, Santa Cruz Biotechnology), Anti-SPRTN antibody (1:500) (6F2^37^), Anti-RNF4 antibody (1:500) (AF7964, R&D systems), Anti-GAPDH (14C10) antibody (1:2000) (2118, Cell Signaling), Anti-Histone H2A antibody (1:1000) (07-146, Merck), Anti-Histone H2B antibody (1:1000) (10799, Cell Signaling), Anti-Histone H3 antibody (1:1000) (4499 S, Cell Signaling), Anti-PIAS4 antibody (1:500) (SC-166744, Santa Cruz Biotechnology), Anti-Flag (1:2000) (F1804, Sigma-Aldrich) Anti-TEX264 (1:500) (sc-100944, Santa Cruz Biotechnology), Anti-Vinculin (1:1000) (sc-73614, Santa Cruz Biotechnology). Following incubation with primary antibody overnight, membranes were washed with TBS-T and incubated for 1 h with corresponding secondary antibodies (Goat Anti-Mouse Immunoglobulins/HRP, P0447, Dako; Swine Anti-Rabbit Immunoglobulins/HRP, P0399, Dako; Goat Anti-Rat Immunoglobulins/HRP, A9037, Sigma; Rabbit Anti-Goat Immunoglobulins/HRP, A8919, Sigma). To help visualize bands, brigthness and contrast of blots were globally adjusted using ImageLab (Bio-Rad) version 5.2. Uncropped scans of all blots are provided in the Source Data file.

### Identification of DNA-protein crosslinks by quantitative proteomics

Agarose plugs were reduced, alkylated and digested with trypsin. The resulting peptides were purified using StageTips and resuspended in 15 µl of 0.1% formic acid solution. For LC-MS/MS purposes, desalted peptides were injected in an Ultimate 3000 RSLCnano system (Thermo) and separated in a 15-cm analytical column (75 μm ID home-packed with ReproSil-Pur C18-AQ 2.4 μm from Dr. Maisch) with a 50-min gradient from 5 to 60% acetonitrile in 0.1% formic acid. The effluent from the HPLC was directly electrosprayed into an LTQ-Orbitrap mass spectrometer XL (Thermo) operated in data dependent mode to automatically switch between full scan MS and MS/MS acquisition. Typical parameters were as follows: survey full scan MS spectra (from m/z 250–1600) were acquired in the Orbitrap with resolution R = 60,000 at m/z 400 (AGC target of 5 × 10^5^). The three most intense peptide ions with charge states between 2 and 4 were sequentially isolated to a target value of 10,000 and fragmented in the linear ion trap by collision induced dissociation (CID). All fragment ion spectra were recorded in the LTQ part of the instrument. For all measurements with the Orbitrap detector, 3 lock-mass ions from ambient air were used for internal calibration. Typical MS conditions were: spray voltage, 1.5 kV; no sheath and auxiliary gas flow; heated capillary temperature, 200 °C; normalized CID energy 35%; activation q = 0.25; activation time = 30 ms. MaxQuant 1.6.6.0 was used to identify proteins and quantify by iBAQ with the following parameters: Database, Uniprot_UP000005604_Hsapiens_20191107; MS tol, 10ppm; MS/MS tol, 0.5 Da; Peptide FDR, 0.1; Protein FDR, 0.01 Min. peptide Length, 7; Variable modifications, Oxidation (M); Fixed modifications, Carbamidomethyl (C); Peptides for protein quantitation, razor and unique; Min. peptides, 1; Minute. ratio count, 2. To identify significantly enriched proteins, MaxQuant output data were further processes in R. LFQ intensity values were log2 transformed. Missing values were imputated based on a probabilistic dropout function using the proDA R-package setting the untreated benzonase condition as a reference level (Ahlmann-Eltze and Anders, 2020). Proteins that were not identified in at least 3 replicates of either non-benzonase treated condition were removed, if they were simultaneously not detected in more than 12 out of 24 samples. Differential abundance of proteins was calculated using a Wald-test with Benjamini Hochberg FDR correction. Identified proteins were considered significantly enriched if their log_2_ fold enrichment was greater than 2 and FDR adjusted *p*-value smaller than 0.01.

### Plasmids and site-directed mutagenesis

pCMV6-RNF4-DDK-Myc was purchased from Origene (#RC207273). pIRES-AcFL was a gift from the Boulton lab. pcDNA5-FRT/TO-YFP-SPRTN-WT-Strep, pcDNA5-FRT/TO-YFP-SPRTN-EQ (E112Q)-Strep pcDNA5-FRT/TO-YFP-SPRTN-ΔC-Strep, pcDNA5-FRT/TO-YFP-SPRTN-PIP*(Y331A/F332A)Strep, pcDNA5-FRT/TO-YFP-SPRTN-SHP*(F253A/L260A)-Strep, pcDNA5-FRT/TO-YFP-SPRTN-ZBD*(R185A)-Strep, pcDNA5-FRT/TO-YFP-SPRTN-UBZ*(D473A)-Strep, pcDNA5-FRT/TO-YFP-SPRTN-UBZ*(D473A)-Ub and pNIC-STREP-ZB-SPRTN-WT have been described previously^13,23,37^. pcDNA5-FRT/TO-YFP-SPRTN-Δ241-400-Strep, pcDNA5-FRT/TO-YFP-SPRTN-Δ241-400-UBZ*-(D473A)-Strep, pcDNA5-FRT/TO-YFP-NLS-SPRTN-ΔC-Strep, pCMV6-RNF4-CS1(C132A/C135A)-DDK-Myc, pNIC-STREP-ZB-SPRTN-UBZ*(D473A), and pNIC-STREP-ZB-SPRTN-ΔC were generated by Q5 site-directed mutagenesis (#E0554S, NEB) according to manufacturer’s instructions. siRNA-resistant variants of pcDNA5-FRT/TO-YFP-SPRTN-WT-Strep and pcDNA5-FRT/TO-YFP-SPRTN-EQ (E112Q)-Strep were generated by Q5 site-directed mutagenesis using primers Oshubo-141 (CGAAAACTATTCAAAAAAAGGCAAAGGAAAG) and Oshubo-142 (GGCTCTTTTATTTTTATGTAAGTGCCTCC) introducing silent mutations in the region targeted by siSPRTN#1. All plasmids generated in this study are available from the corresponding author upon request.

### Cell viability and drug sensitivity

To measure formaldehyde sensitivity, 500 cells were seeded per well in triplicates in 6-well plates. The next day, cells were treated with the indicated doses formaldehyde for 1 h followed by two washes with PBS. After 7 days, cells were stained with crystal violet.

To measure 5-azadC sensitivity of HeLa WT and *RNF4* KO cells, 5 × 10^3^ cells were seeded in technical quadruplicates in 24-well plates. 5-azadC was added at the indicated concentration 16 h after seeding. After 96 h, cell viability was measured by AlamarBlue assay (Resazurin, R7017, Sigma). To determine complementation of 5-azadC sensitivity, HeLa WT and *RNF4* KO cells were transfected with pIRES-AcFL (expressing GFP-Flag), pCMV6-RNF4-DDK-Myc, or pCMV6-RNF4-CS1-DDK-Myc (CS1, C132A/C135A variant as in^48^) plasmids. 3 µg of plasmid were mixed with 100 µl of Opti-MEM Medium and incubated for 5 min. 3 µl of Lipofectamine 2000 were mixed with 100 µl of Opti-MEM Medium and incubated for 5 min. After incubation, both solutions were mixed and incubated for additional 15 min. The solution was then added to the cells in 800 µl of media. 5 × 10^3^ transfected cells were then re-seeded in technical quadruplicates in 24-well plates. 5-azadC was added at the indicated concentration 16 h after seeding. After 96 h, cell viability was measured by AlamarBlue assay.

For viability and 5-azadC-sensitivity measurements of HeLa T-Rex Flp-In (WT, *SPRTN-ΔC, AAVS1 #1, SPRTN-ΔUBZ #3 and SPRTN-ΔUBZ #10*), HeLa (WT and *RNF4* KO) cells, and U2OS T-Rex Flp-In (WT, *SPRTN-ΔC* #2, *SPRTN-ΔC* #4, *SPRTN-ΔUBZ #10* and *SPRTN-ΔUBZ #12*), 2 × 10^5^ cells were seeded in 6-well plates, followed by transfection with siRNAs, as indicated in figure legends. To measure cell viability, transfected cells were re-seeded the following day (5 × 10^3^ cells for HeLa T-Rex Flp-In and HeLa cells; 1 × 10^4^ cells for U2OS T-Rex Flp-In cells) in technical triplicates in 12-well plate. Cell confluency was monitored and analysed using a IncuCyte S3 live cell imaging system every 12 h for 5 days. Following imaging, cells were stained with crystal violet. To measure 5-azadC sensitivity, 5 × 10^3^ cells were re-seeded the day after transfection in technical quadruplicates in 24-well plates. 5-azadC was added at the indicated concentration 16 h after seeding. After 96 h, cell viability was measured by AlamarBlue assay.

### DNA and RNA synthesis measurements

1 × 10^6^ cells were seeded in 6-cm dishes. The next day, cells were pre-treated with flavopiridol (10 µM, 1 h), aphidicolin (3 µM, 2 h) or palbociclib (5 µM (Sigma, PZ0383), 48 h), as indicated in figure legends. To measure RNA synthesis, 400 µM 5-ethynyl-uridine (EU, Jena Bioscience, CLK-N002-10) was then added for 30 min. To measure DNA synthesis, 100 µM 5-ethynyl-2’-deoxyuridine (EdU, Jena Bioscience, CLK-N001-100) was added for 30 min. Next, cells were washed twice with PBS, harvested by trypsinization and stained with eFluor780 viability dye (Thermo, 65-0865-14) for 30 min at 4 °C. Next, pellets were washed with PBS containing 1% BSA and fixed with 4% formaldehyde for 15 min. Following another wash, cells were permeabilized by 0.25% Triton-X in PBS, washed, and incubated with Click-it mix (Tris, 39.5 mM, pH8; Alexa Fluor 488-Azide (Thermo, B40953), 0.06 mM; CuSO4, 4 mM; Ascorbic Acid, 40 µg/ml; DAPI 1 µg/ml) for 30 min. After a final wash step, cells were resuspended in PBS containing 1% BSA and analyzed on a BD LSRFortessa (BD Bioscience) equipped with 355/405/488/561/640 nm lasers with a minimum count of 10,000 events. The mean fluorescence intensity (MFI) in FITC channel was measured excluding dead cells (eFluor780 stained) and the results were analyzed using FlowJo™ v10.8.1 Software (BD Life Sciences). In brief, cell debris and aggregates were excluded by SSC-A/FSC-A and single cells were gated by SSC-H/SSC-A. Live cells were identified by SSC-A/APC-Cy7-A and the fraction of EdU/EU positive cells was identfied based on FITC-A fluorescence intensity (Supplementary Fig. 8).

### Immunofluorescence staining

Doxycycline (1 µg/ml, D9891, Sigma) was added overnight to HeLa-TREx Flp-in cells to induce expression of SPRTN variants. The next day, cells were fixed in 4% formaldehyde (28906, Thermo Scientific) followed by permeabilizing and blocking with PBGT buffer (1X PBS, 0.2% fish skin gelatin, 0.5% BSA, 0.5% Triton X-100) (45 min at room temperature) and then incubated with anti-GFP antibody (Chromotek, PABG1) for 1 h at room temperature. Coverslips were washed 3 times for 5 min with PBGT buffer and incubated with Alexa Fluor 488 goat anti-mouse secondary antibody (A-11001, Thermo Scientific) and DAPI (0.5 μg/ml, 62248, Thermo Fisher) for 1 h at room temperature. Coverslips were mounted in Prolong Gold Antifade Mountant (P10144, Thermo Fisher) and images were acquired using a ZEISS LSM710 confocal microscope and software ZEN 2009 (Carl Zeiss) version 5.5.0.443. Image processing was done using ImageJ (v1.53t).

### Recombinant protein purification

Recombinant SPRTN (WT, EQ (E112Q), UBZ* (D473A)) protein was expressed in *E. coli BL21* and purified as previously described^23^. The protocol was slightly modified for the purification of SPRTN-ΔC, for which the N-terminal Strep-Zb-tag was removed using a Strep-tagged TEV protease. Tag and TEV protease were removed by applying the sample to Strep-Tactin®XT Superflow® high capacity cartridges, before the collected flow-through was further purified by size exclusion chromatography.

### In vitro SPRTN autocleavage

Reactions were performed for 2 h at 25 °C in 20 μL containing 2 µM recombinant SPRTN and 11.14 nM circular single-stranded DNA (ΦX174 Virion DNA, #N3023, NEB). The reaction buffer comprised 19.5 mM HEPES/KOH pH 7.2, 2.9% glycerol, 80 mM KCl, and 4.95 mM TCEP. Reactions were stopped by 4 x LDS sample buffer supplemented with 5% β-mercaptoethanol and boiling at 95 °C for 10 min, and resolved on SDS-PAGE gels (4–12% Bis-Tris) using MOPS buffer and stained with SYPRO Ruby (#S12000, Thermo Fisher Scientific) following manufacturer’s instructions. Gels were photographed using a BioRad Chemidoc MP system and cleavage was quantified using ImageJ (v1.53t). The fraction of cleaved recombinant SPRTN was calculated by dividing the amount of remaining full-length protein in the presence of DNA by the amount of full-length protein in the absence of DNA.

### SPRTN autocleavage in cells

1 × 10^5^ cells per well were seeded in 12-well plates in the evening. The next morning cells were pre-treated or not with aphidicolin (3 µM) for 2 h and then in combination with formaldehyde (250 µM), CPT (500 nM) or etoposide (50 µM, Sigma, 341205). In the indicated timepoint, cells were washed with PBS 1x and resuspended in 1x LDS. The samples were then boiled and resolved in 4–12% 20-well gels. For palbociclib treatment, cells were seeded in 10 cm dishes in the presence of palbociclib (5 µM), reseeded in palbociclib-containing medium after 30 h in 12-well plates, before autoclevage was induced as described above.

### Protein G-oligonucleotide conjugation

Protein G-oligonucleotide conjugates were generated as previously described^23,44^. In brief, Protein G (#6510, BioVision) was conjugated to oligonucleotide X15 (5’−6-FAM-ACC AGT GCC TTG CT[SH-C9-dT] GGA CAT CTT TGC CCA-3’) (Ella BioTech GmbH), which contained a 6-FAM label at the 5’-end and a phosphate group at the 3’-end. Conjugation was performed using the proFIRE Amine Coupling Kit (Dynamic Biosensors) and the conjugate purified with the proFIRE device (Dynamic Biosensors) through ion exchange chromatography. The conjugate concentration was determined by measuring 6-FAM fluorescence in a Tecan Spark plate reader using a NanoQuant plate. For Protein G-oligonucleotide conjugate cleavage assays, conjugates were annealed to a 2x excess of complementary reverse oligonucleotide oDY_72 (5’-TGGGCAAAGATGTCC-3’) forming a single-/double-stranded DNA junction.

### Protein G-oligonucleotide conjugate cleavage assay

Cleavage of Protein G-oligonucleotide conjugates by SPRTN was performed in a reaction containing increasing concentrations of SPRTN (2, 10 and 50 nM) and 10 nM conjugate (or free DNA as control) in a final reaction buffer of 17.5 mM HEPES/KOH pH 7.2, 85 mM KCl, 3.5% glycerol, 5.5 mM TCEP and 0.12 mg/mL BSA. Reactions were incubated for 2 h at 25 °C. Unstained urea loading dye (15% Ficoll®, 8 M Urea) was added and reactions were resolved on 8 M Urea, 15% Acrylamide, 1x TBE gels using 1x TBE as running buffer. Gels were photographed using a BioRad Chemidoc MP system and cleavage was quantified using ImageJ (v1.53t) by calculating the ratio between cleaved and total conjugate.

### Reporting summary

Further information on research design is available in the Nature Portfolio Reporting Summary linked to this article.

### Supplementary information

Supplementary Information

Description of Additional Supplementary Files

Supplementary Data 1

Supplementary Data 2

Supplementary Data 3

Reporting Summary

### Source data

Source Data

## Data Availability

Mass spectrometry data have been deposited to the ProteomeXchange Consortium via the PRIDE partner repository with the dataset identifier PXD026654^49^. Database Uniprot_UP000005604_Hsapiens_20191107 was used to identify proteins. Source data are provided with this paper. All other data that support this study are available from the corresponding author upon request. Source data are provided with this paper.
