# Supplementary material for: SPRTN patient variants cause global-genome DNA-protein crosslink repair defects
Source: Nat Commun. 2023 Jan 21;14:352. doi: 10.1038/s41467-023-35988-1 (PMC9867749; doi:10.1038/s41467-023-35988-1)

Supplementary Information

SPRTN patient variants cause global-genome DNA-protein crosslink repair defects

Pedro Weickert[1,2,4], Hao-Yi Li[1,2,4], Maximilian J. Götz[1,2], Sophie Dürauer[1,2], Denitsa Yaneva[1,2], Shubo Zhao[1,2], Jacqueline Cordes[1,2], Aleida C. Acampora[1,2], Ignasi Forne[3], Axel Imhof[3], and Julian Stingele[1,2,*]

[1]Department of Biochemistry, Ludwig-Maximilians-University, 81377 Munich, Germany.

[2]Gene Center, Ludwig-Maximilians-University, 81377 Munich, Germany.

[3]Protein Analysis Unit (ZfP), BioMedical Center (BMC), Ludwig-Maximilians-University, 82152 Martinsried, Germany.

[4]These authors contributed equally: Pedro Weickert, Hao-Yi Li

*Correspondence: stingele@genzentrum.lmu.de

Extended Data Fig. 1. Analysis of DNA-protein crosslink formation by Purification of x-linked Proteins (PxP) assay. a InstantBlue-stained SDS-PAGE gel used for electro-elution of camptothecin (CPT)-treated PxP samples shown in Fig. 1b. Two plugs per condition were used. **b** InstantBlue-stained SDS-PAGE gel used for electro-elution of formaldehyde (FA)-treated PxP samples shown in Fig. 1c. Two plugs per condition were used. **c** HeLa cells were treated with the indicated concentrations of FA for 1 hour, followed by growth in drug-free medium for 7 days and staining by crystal violet. Source data are provided as a Source Data file.

Extended Data Figure 1

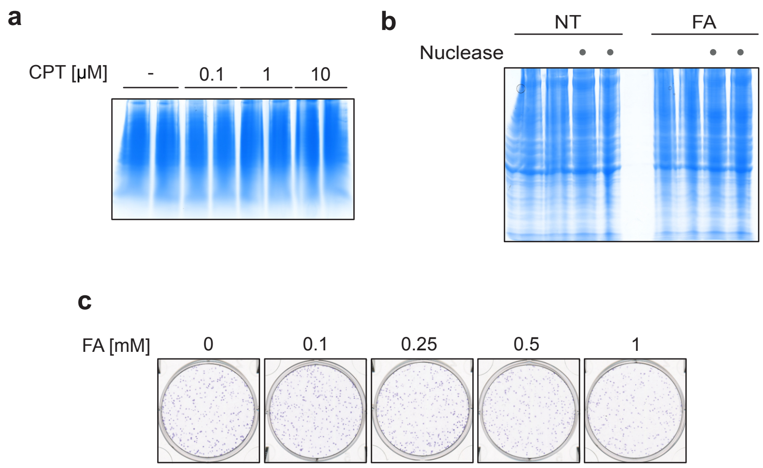

Extended Data Fig. 2. Analysis of 5-azadC-induced DNMT1-DPC repair by PxP assay. a HeLa T-REx Flp-In cells were treated as depicted in Fig. 2a and harvested directly after 5-azadC exposure. DPCs were isolated using PxP including a nuclease treatment prior to electro-elution as indicated, and analysed by western blotting using the indicated antibodies. This control experiment was performed once. **b** InstantBlue-stained SDS-PAGE gel used for electro-elution of 5-azadC-treated PxP samples shown in (**a**). Two plugs per condition were used. **c** HeLa T-REx Flp-In cells transfected with the indicated siRNAs were treated and analysed as depicted in Fig. 2a. DPCs were isolated using PxP and analysed by western blotting using the indicated antibodies. **d** HeLa T-REx Flp-In cells were treated as in Fig. 2e with the difference that ubiquitylation inhibitor (Ub-E1i TAK-243, 1 µM) was added already 1 hour prior to addition of 5-azadC (top). DPCs were isolated using PxP and analysed by western blotting using the indicated antibodies (bottom). **e** 5-azadC-induced DNMT1-DPC formation and repair upon inhibition of p97 (p97i CB-5083, 5µM). HeLa T-REx Flp-In cells were treated as depicted in Fig. 2a prior to extraction of DPCs using PxP and analysis of samples using western blotting using the indicated antibodies. **f** HeLa T-REx Flp-In cells transfected with the indicated siRNAs were treated and analysed as depicted in Fig. 2a. DPCs were isolated using PxP and analysed by western blotting using the indicated antibodies. **g** U2OS T-REx Flp-In WT and *PIAS4* knock-out (KO) cells were treated and analysed as shown in Fig. 2a. DPCs were isolated by PxP and analysed by western blotting using the indicated antibodies. Experiments were repeated three (**c-f**) or two **(g)** times with similar results. Source data are provided as a Source Data file.

Extended Data Figure 2

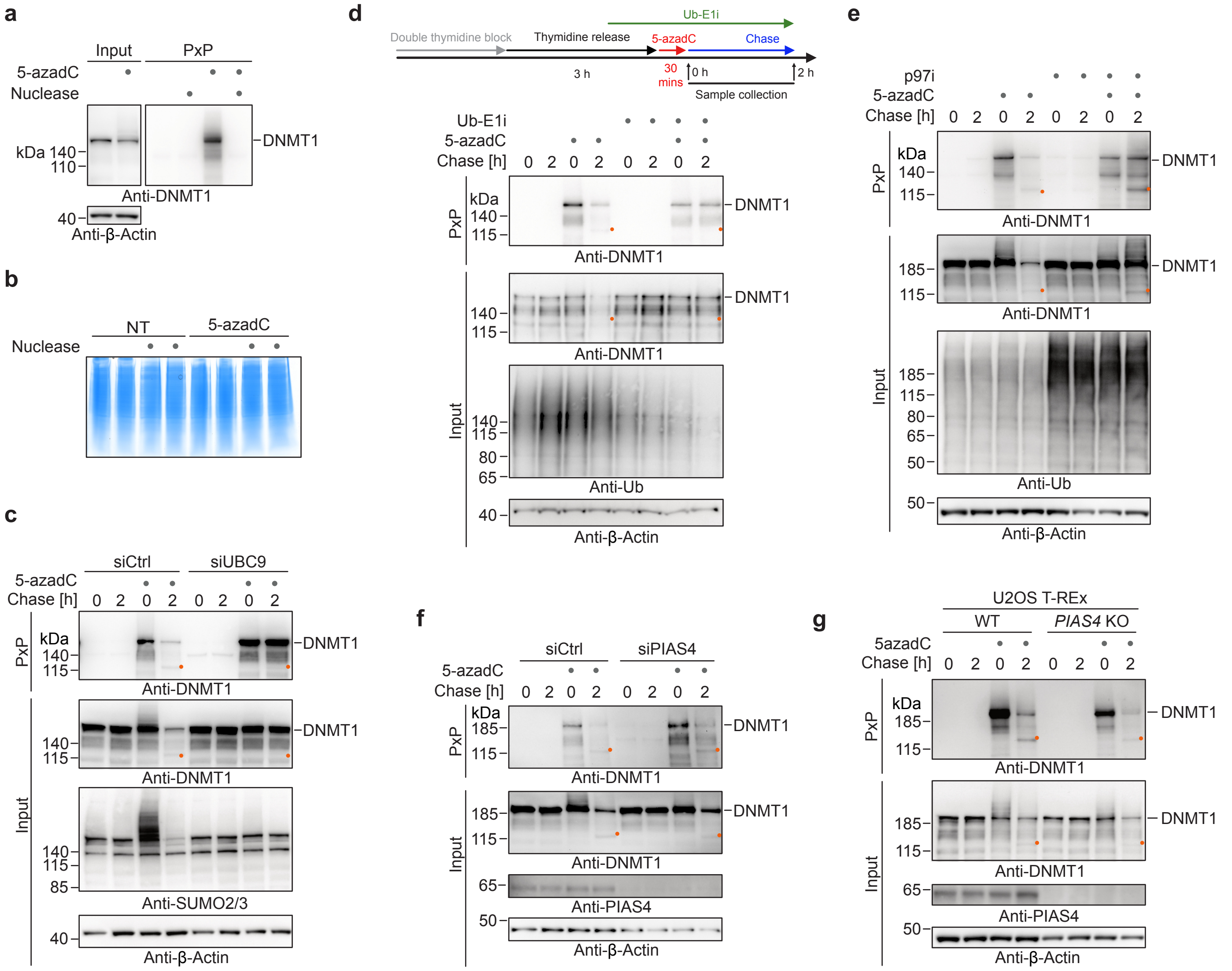

Extended Data Fig. 3. Analysis of 5-azadC-induced DNMT1-DPC repair upon RNF4 loss. a Western blot analysis of HeLa WT and *RNF4* knock-out (KO) cells (left). Cells were treated with increasing concentrations of 5-azadC for 96 hours prior to assessment of viability by AlamarBlue assay. Values represent the mean ± SD of 4 technical replicates normalized to untreated cells (right). **b** Western blot analysis of HeLa WT and *RNF4* KO cells transfected with plasmids encoding AcGFP-Flag or C-terminally Flag-tagged RNF4 (wildtype (WT) or catalytically inactive variant (CS1, C132A/C135A)) (left). Cells were treated with increasing concentrations of 5-azadC for 96 hours prior to assessment of viability by AlamarBlue assay. Values represent the mean ± SD of 4 technical replicates normalized to untreated cells (right). **c** HeLa WT and *RNF4* KO cells were treated and analysed as depicted including an optional treatment with ubiquitylation inhibitor (Ub-E1i TAK-243, 1 μM) (top), prior to extraction of DPCs using PxP and analysis of samples by western blotting using the indicated antibodies (bottom). The experiment was repeated three times with similar results. Source data are provided as a Source Data file.

Extended Data Figure 3

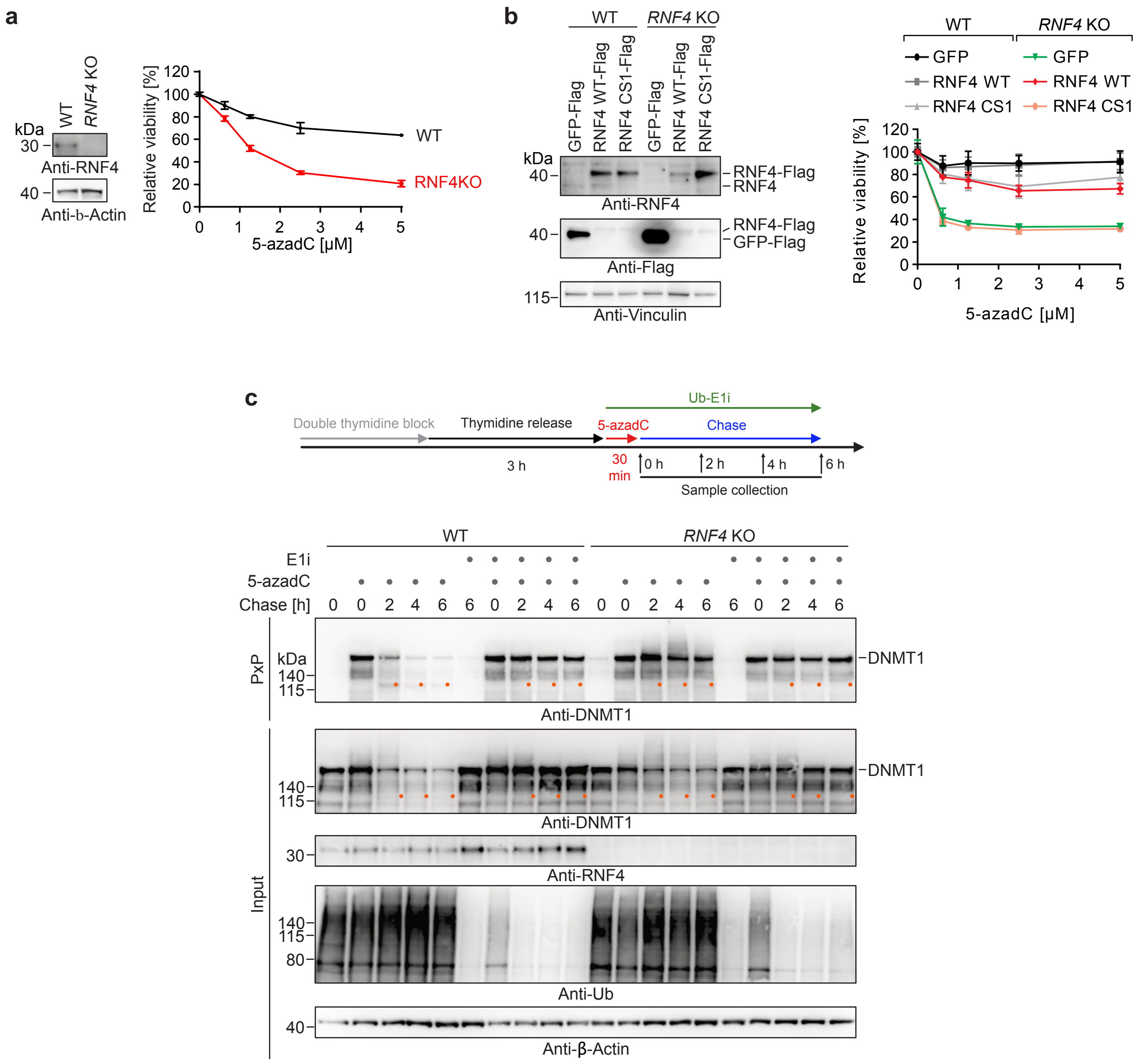

Extended Data Fig. 4. Analysis of DPC-induced SPRTN activation. a HeLa T-REx Flp-In cells were treated with aphidicolin (3 μM) for 2 hours and then incubated with EdU-containing medium for 30 min. EdU incorporation was quantified by flow cytometry. **b** Polyclonal pools of HeLa T-REx Flp-In cells transfected with Cas9 and sgRNAs targeting the safe-harbour site AAVS1 or the TEX264 locus were treated as depicted in Fig. 2a. DPCs were isolated using PxP and analysed by western blotting using the indicated antibodies. The experiment was repeated three times with similar results. **c-d** 5-azadC-induced DNMT1-DPC repair upon inhibition of transcription assessed by PxP. HeLa T-REx Flp-In cells were treated as depicted including an addition of flavopiridol (10 μM), as indicated. EU incorporation (30 min) was quantified by flow cytometry after a 1-hour flavopiridol pre-treatment (**c**). DNMT1-DPCs were isolated using PxP and analysed by western blotting using the indicated antibodies (**d**). The experiment was repeated twice with similar results. **e** HeLa cells were treated with aphidicolin (3 μM) for 2 hours and then incubated with EdU-containing medium for 30 min. EdU incorporation was quantified by flow cytometry. **f** U2OS T-REx Flp-In cells were treated with palbociclib (5 μM) for 48 hours and then incubated with EdU containing medium for 30 min. EdU incorporation was quantified by flow cytometry. **g-i** U2OS T-REx Flp-In cells were treated with palbociclib (5 μM) for 48 hours and then treated with FA (250 μM) (**g**), CPT (500 nM) (**h**) or ETO (50 μM) (**i**) as indicated, before SPRTN autocleavage was assessed by Western blotting. Experiments shown in (**g-i)** were repeated three times with similar results. Source data are provided as a Source Data file.

Extended Data Figure 4

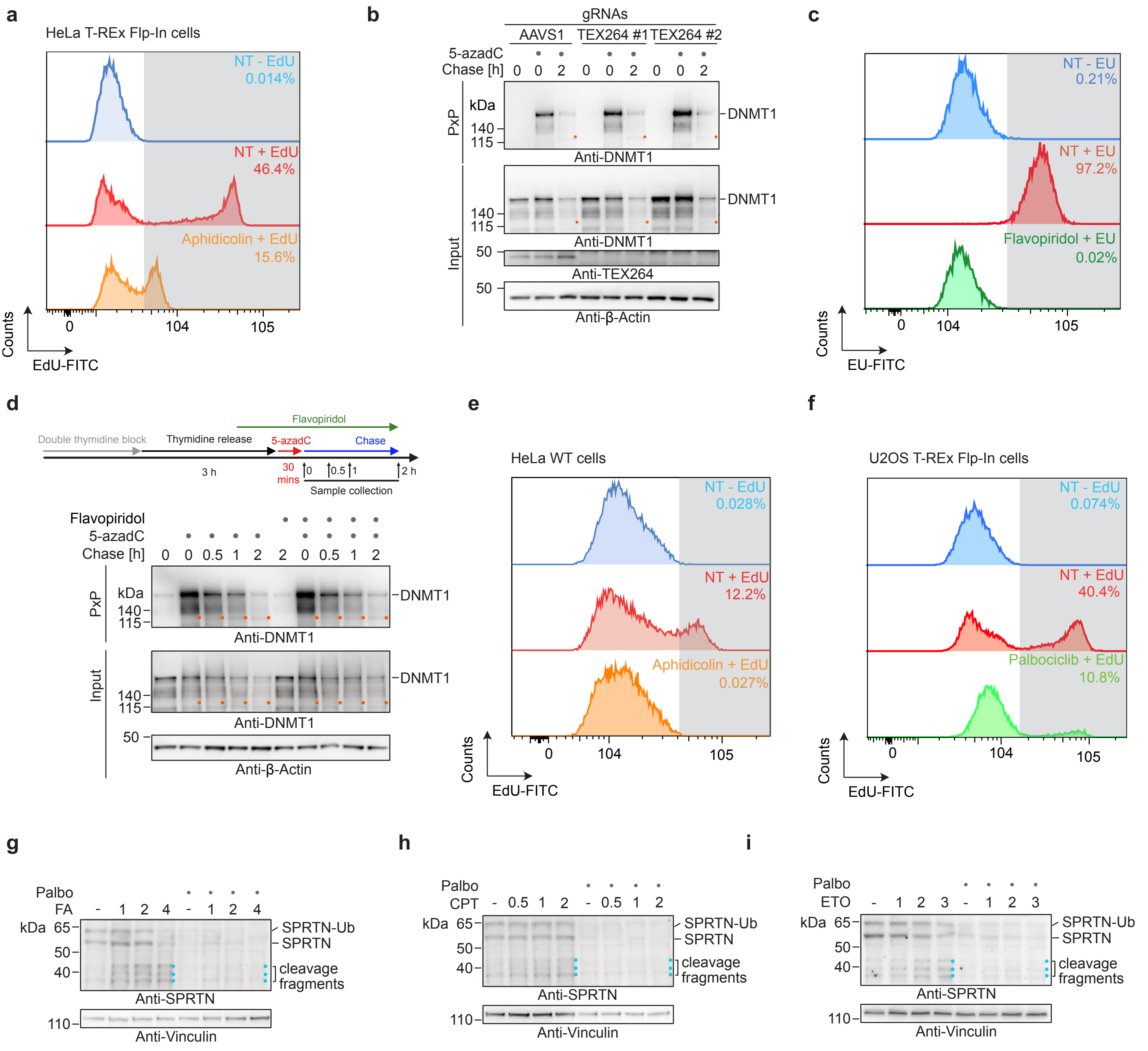

Extended Data Fig. 5. Generation and characterization of *SPRTN-ΔC* cells. a Depiction of the editing strategy for the generation of HeLa T-REx Flp-In *SPRTN-ΔC* cells and Sanger sequencing traces of both edited alleles. **b-e** 5-azadC sensitivity and viability of HeLa T-REx Flp-In WT, *SPRTN-ΔC*, or HeLa WT, *RNF4* knock-out (KO) cells were analysed 72 hours after transfection with the indicated siRNAs. Western blot analysis of HeLa T-REx Flp-In WT, *SPRTN-ΔC* (**b**), or *RNF4* KO cells (**d**) transfected with the indicated siRNAs (left). Transfected cells were exposed to increasing concentrations of 5-azadC for 96 hours, prior to assessment of cell viability using AlamarBlue assay (right). Values represent the mean ± SD of 3 biological replicates normalized to untreated cells. HeLa T-REx Flp-In WT, *SPRTN-ΔC* (**c**), or HeLa WT, *RNF4* KO cells (**e**) were transfected with the indicated siRNAs and cell confluency was monitored over 5 days by IncuCyte live cell imaging every 12 hours (left, values represent the mean ± SD of 3 technical replicates), before cells were stained with crystal violet (right). **f** Depiction of the editing strategy for the generation of U2OS T-REx Flp-In *SPRTN-ΔC* cells and Sanger sequencing traces of edited alleles of two different clones. **g** U2OS T-REx Flp-In WT and *SPRTN-ΔC* cells were treated as depicted in Fig. 2a but with a 1-hour 5-azadC incorporation, before DPCs were isolated by PxP and analysed by western blotting using the indicated antibodies. The experiment was repeated twice with similar results. **h** Western blot analysis of U2OS T-REx Flp-In WT and *SPRTN-ΔC* cells transfected with the indicated siRNAs used for viability assays shown in Fig. 4e. Knock-down efficiency was confirmed twice with similar results. Source data are provided as a Source Data file.

Extended Data Figure 5

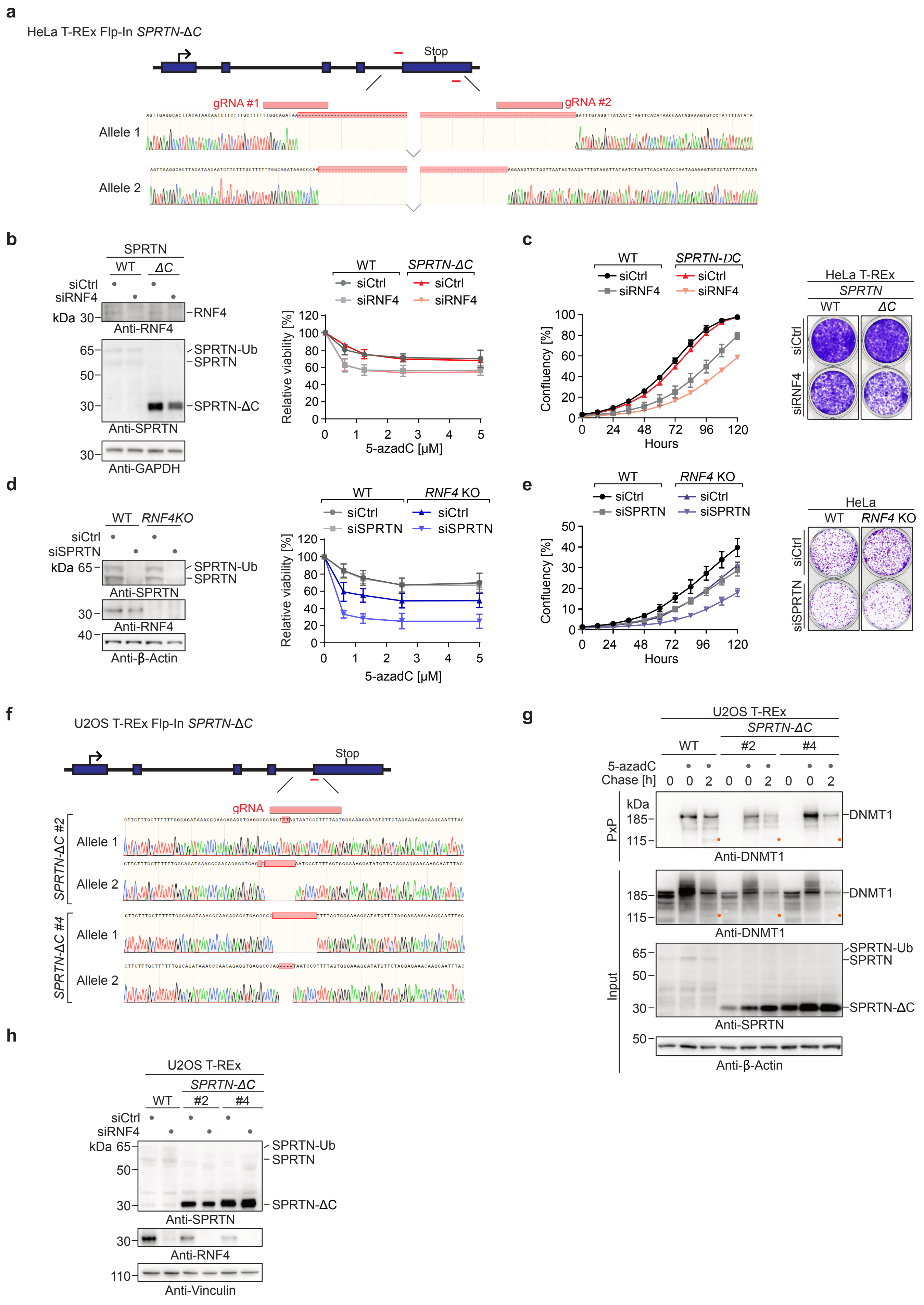

Extended Data Fig. 6. Structure-function-analysis of DPC cleavage by SPRTN. a HeLa T-REx Flp-In *SPRTN-ΔC* cells complemented with variants bearing amino acid replacements in SPRTN's interaction motifs/domains for binding to p97 (SHP*), PCNA (PIP*) and ubiquitin (UBZ*) were treated as depicted in Fig. 2a. DPCs were isolated using PxP and analysed by western blotting using the indicated antibodies. The experiment was repeated three times with similar results. **b** HeLa T-REx Flp-In *SPRTN-ΔC* cells were complemented with YFP, YFP-SPRTN-Strep, YFP-SPRTN-EQ-Strep, YFP-SPRTN-UBZ*-Strep and YFP-SPRTN-UBZ*-Ub (ubiquitin C-terminal fusion) and treated as shown in Fig. 2a, before DPCs were isolated using PxP and analysed by western blotting using the indicated antibodies. The experiment was repeated twice with similar results. **c** Protein G covalently conjugated to a fluorescently-labelled single-/double-stranded DNA junction was incubated for 2 hours with increasing concentrations of the indicated recombinant SPRTN variants *in vitro*, followed by analysis using denaturing Urea-PAGE (top). Quantifications show the mean ± SD of 3 independent experiments (bottom). **d** SPRTN autocleavage activity was assessed by incubating the indicated recombinant SPRTN variants in the presence or absence of DNA (ΦX174 Virion) for 2 hours, prior to analysis of cleavage using SYPRO Ruby-stained SDS-PAGE (left). Asterisks indicate SPRTN autocleavage fragments. Quantifications show the mean ± SD of 3 independent experiments (right). Source data are provided as a Source Data file.

Extended Data Figure 6

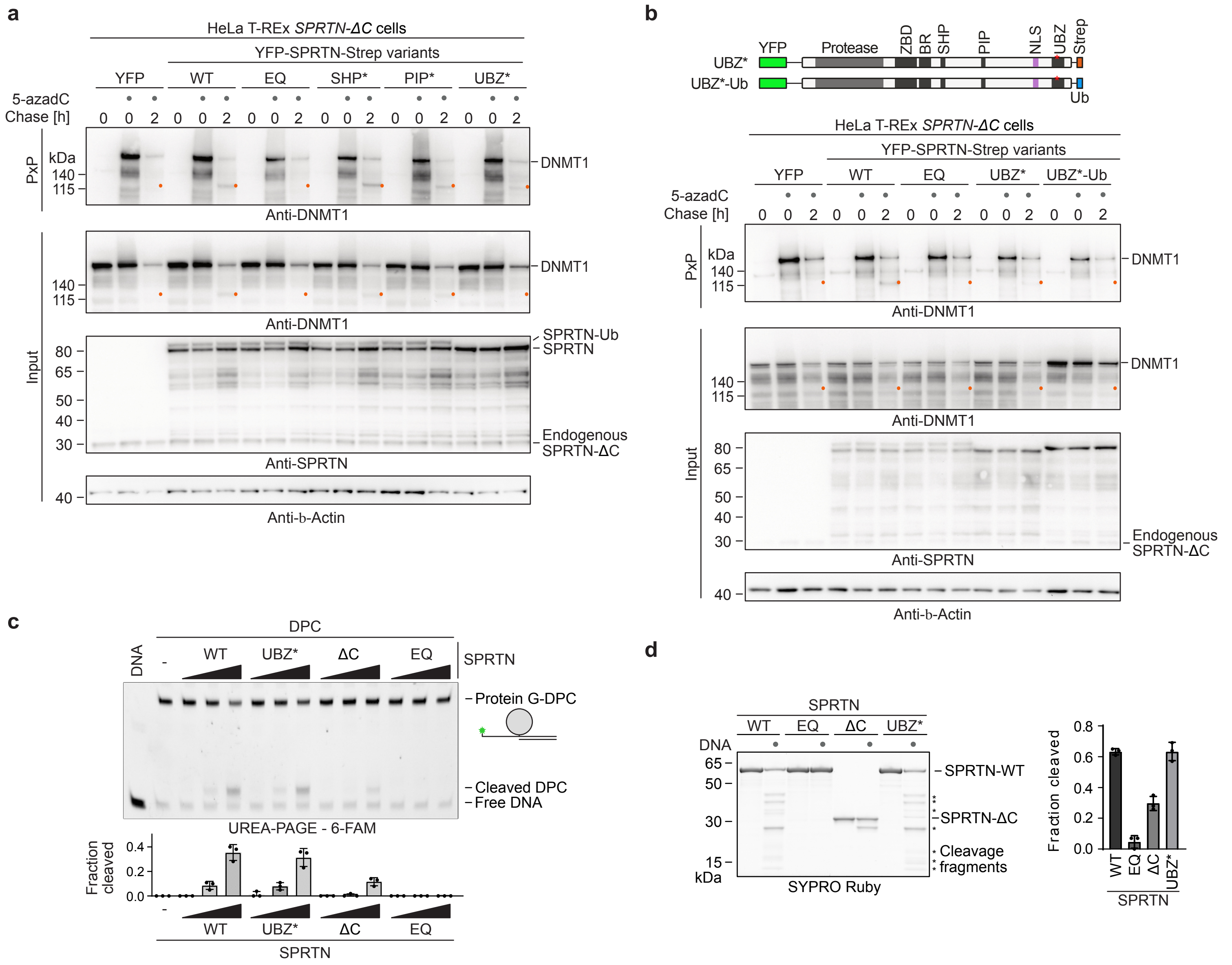

Extended Data Fig. 7. Generation and characterization of *SPRTN-ΔUBZ* cells a Depiction of the editing strategy for the generation of HeLa T-REx Flp-In *SPRTN-ΔUBZ* cells and sanger sequencing traces of the edited alleles of two different clones (top). Amino acid sequences encoded by edited *SPRTN-ΔUBZ* alleles. The sequence of the SPRTN-UBZ domain is highlighted in grey. Residues coordinating the zinc ion of the UBZ are indicated with asterisks. Aspartate D473, which is essential for ubiquitin binding is indicated with # (bottom). **b-d** HeLa T-REx Flp-In WT or *SPRTN-ΔUBZ* #3 cells were treated with formaldehyde (FA, 250 μM) (**b**), CPT (500 nM) (**c**) or ETO (50 μM) (**d**) as indicated in the absence or presence of aphidicolin including a 2 hours pre-treatment. The whole cell lysates were harvested for analysis of SPRTN autocleavage by western blotting using the indicated antibodies. **(b-d)** were repeated three times with similar results. **e** Western blot analysis of HeLa T-REx Flp-In WT and *SPRTN-ΔUBZ* cells transfected with the indicated siRNAs (top). Transfected cells were exposed to increasing concentrations of 5-azadC for 96 hours, prior to assessment of cell viability using AlamarBlue assay (bottom). Values represent the mean ± SD of 3 biological replicates normalized to untreated cells. **f** HeLa T-REx Flp-In WT and *SPRTN-ΔUBZ* cells were transfected with the indicated siRNAs. Cell confluency was monitored over 5 days by IncuCyte live cell imaging every 12 hours (top, values represent the mean ± SD of 3 technical replicates), before cells were stained with crystal violet (bottom). **g** Western blot analysis of U2OS T-REx Flp-In WT and *SPRTN-ΔUBZ* cells transfected with the indicated siRNAs and used for viability assays shown in Fig. 5e. Knock-down efficiency was confirmed twice with similar results. Source data are provided as a Source Data file.

Extended Data Figure 7

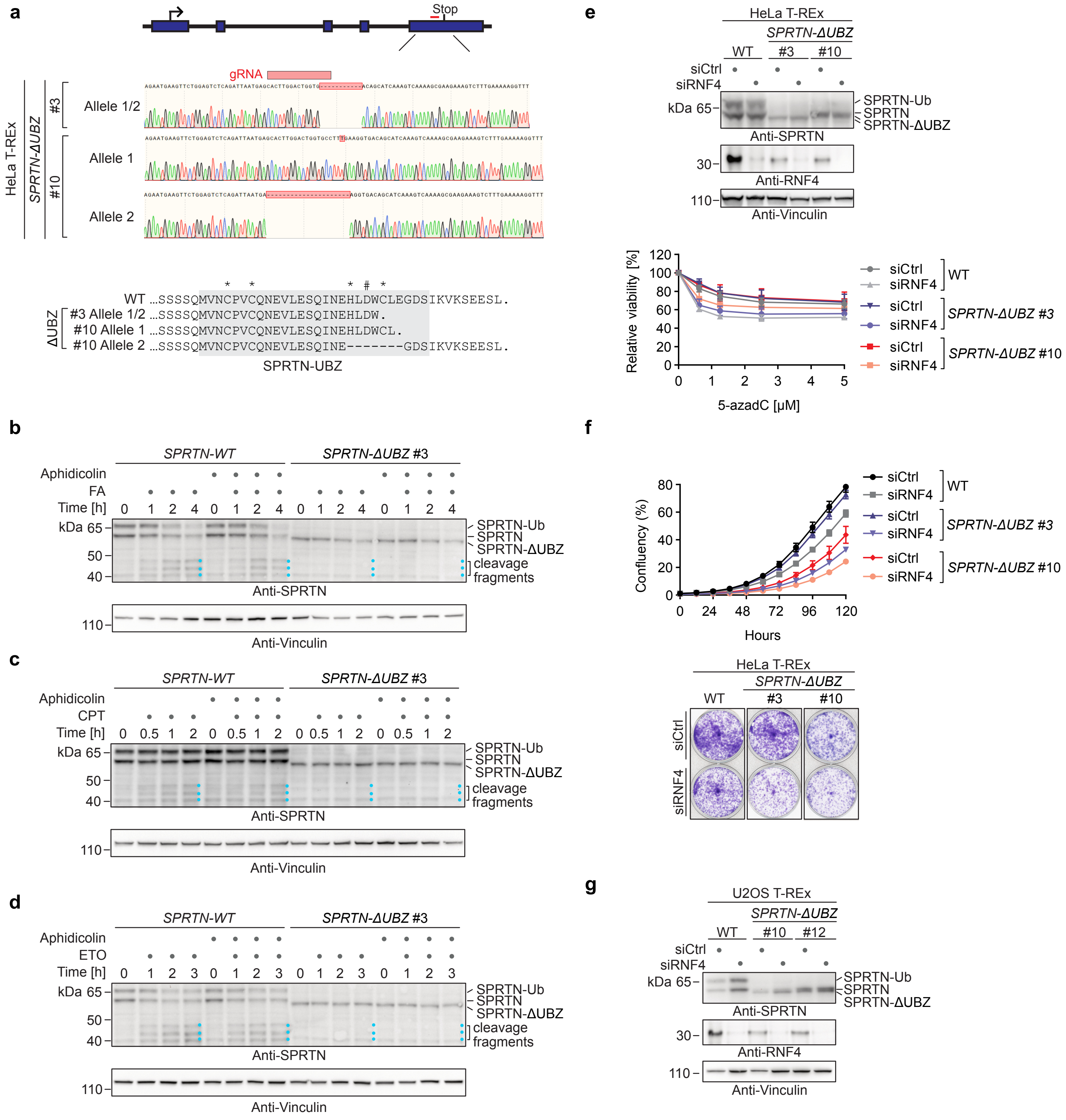

Extended Data Fig. 8. Gating strategy for flow cytometry analysis. Cell debris and aggregates were excluded by SSC-A/FSC-A and single cells were gated by SSC-H/SSC-A. Live cells were identified by SSC-A/APC-Cy7-A and the fraction of EdU/EU positive cells was identified based on FITC-A fluorescence intensity relative to unlabeled cells.

Extended Data Figure 8

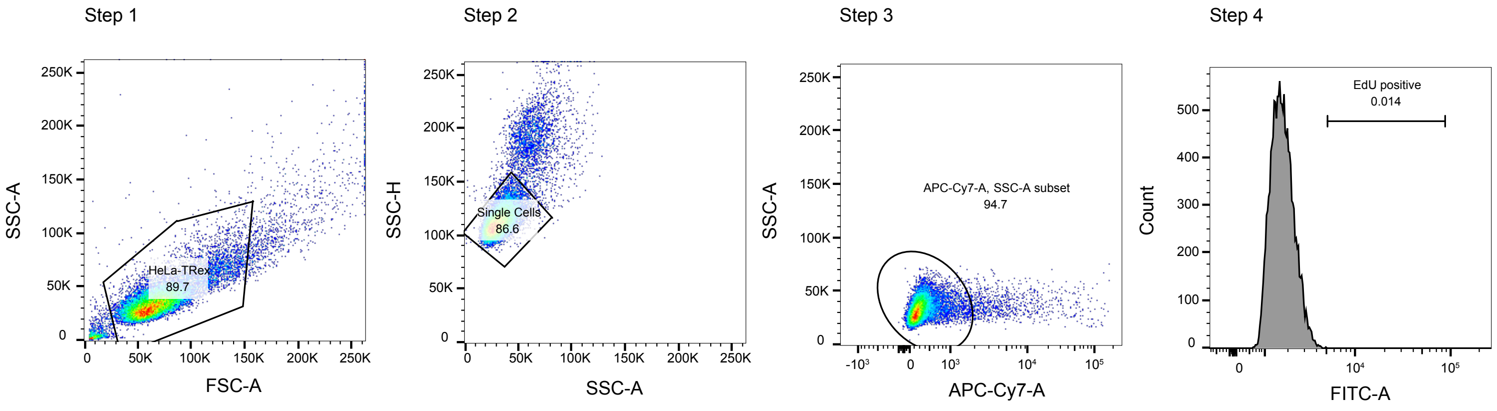

Supplement: Supplementary file 1 — Supplementary Information [file 41467_2023_35988_MOESM1_ESM.pdf]
