# Supplementary material for: SPRTN patient variants cause global-genome DNA-protein crosslink repair defects
Source: Nat Commun. 2023 Jan 21;14:352. doi: 10.1038/s41467-023-35988-1 (PMC9867749; doi:10.1038/s41467-023-35988-1)
Supplement: Supplementary file 2 — Description of Additional Supplementary Files [file 41467_2023_35988_MOESM2_ESM.pdf]

Description of Additional Supplementary Files

File Name: Supplementary Data 1
Description: Statistical test results of formaldehyde treated vs untreated samples generated by the test_diff function of the proDA package, depicted in Fig. 1d. The table contains: The Uniprot identifier (UniprotID), HGNC gene symbol (Gene name), p-value (pval), -log10-FDR adjusted p-value (adj_pval), log2 fold change (diff), standard error (se), degrees of Freedom (df), number of samples observed (n_obs), approximated information available for estimating the protein features as a multitude in one sample (n_approx), indicator whether a protein scored as significant (sig, TRUE or FALSE) as well as the t-statistic (fold change divided by standard error, indicated as t_statistic) and the average abundance.

File Name: Supplementary Data 2
Description: Log2-transformed normalised intensities of all proteins measured and depicted in Fig. 1d-e. The file contains: The Uniprot identifier (UniprotID), HGNC gene symbol (Gene name), the normalised log2-transformed intensity in each replicate of the respective conditions (FA_Rn: formaldehyde-treated replicate n, FABenzCrtl_Rn: Formaldehyde-treated replicate n nuclease control, Untr_Rn: untreated replicate n and UntrBenzCrtl_Rn: untreated replicate n nuclease control. n indicates the replicate numbers 1-6). NA accounts for non-detected.

File Name: Supplementary Data 3
Description: Primers used for genotyping.
