# Supplementary material for: SPRTN patient variants cause global-genome DNA-protein crosslink repair defects
Source: Nat Commun. 2023 Jan 21;14:352. doi: 10.1038/s41467-023-35988-1 (PMC9867749; doi:10.1038/s41467-023-35988-1)
Supplement: Supplementary file 6 — Reporting Summary [file 41467_2023_35988_MOESM6_ESM.pdf]

nature portfolio

Corresponding author(s): Julian Stingele

Last updated by author(s): 2022.12.21

Reporting Summary

Nature Portfolio wishes to improve the reproducibility of the work that we publish. This form provides structure for consistency and transparency in reporting. For further information on Nature Portfolio policies, see our Editorial Policies and the Editorial Policy Checklist.

Statistics

For all statistical analyses, confirm that the following items are present in the figure legend, table legend, main text, or Methods section.

n/a	Confirmed	
☐	☒	The exact sample size (*n*) for each experimental group/condition, given as a discrete number and unit of measurement
☐	☒	A statement on whether measurements were taken from distinct samples or whether the same sample was measured repeatedly
☐	☒	The statistical test(s) used AND whether they are one- or two-sided<br>*Only common tests should be described solely by name; describe more complex techniques in the Methods section.*
☒	☐	A description of all covariates tested
☐	☒	A description of any assumptions or corrections, such as tests of normality and adjustment for multiple comparisons
☐	☒	A full description of the statistical parameters including central tendency (e.g. means) or other basic estimates (e.g. regression coefficient) AND variation (e.g. standard deviation) or associated estimates of uncertainty (e.g. confidence intervals)
☐	☒	For null hypothesis testing, the test statistic (e.g. *F*, *t*, *r*) with confidence intervals, effect sizes, degrees of freedom and *P* value noted<br>*Give P values as exact values whenever suitable.*
☐	☒	For Bayesian analysis, information on the choice of priors and Markov chain Monte Carlo settings
☒	☐	For hierarchical and complex designs, identification of the appropriate level for tests and full reporting of outcomes
☒	☐	Estimates of effect sizes (e.g. Cohen's *d*, Pearson's *r*), indicating how they were calculated

Our web collection on statistics for biologists contains articles on many of the points above.

Software and code

Policy information about availability of computer code

Data collection	ImageLab (Bio-Rad) Version 5.2<br>Ultimate 3000 RSLCnano system (Thermo)<br>Microscopy: Carl Zeiss LSM710<br>ZEN 2009 (Carl Zeiss) Version 5.5.0.443<br>FlowJo Version 10.8.1
Data analysis	GraphPad Prism Version 9.1.0<br>ImageJ v1.53t<br>Raw mass spectrometry data was processed using MaxQuant software suite (v.1.6.6.0, open source).<br>Statistical analysis was performed in the R-studio programming environment (v1.4.1717-3, open source).<br>R version 3.6.2 (2019.12.12, open source).<br>proDA R-package (v1.1.2, open source).<br>Inferred priors using proDA:<br>location prior mean: 22.4<br>location prior scale: 15.4<br>variance prior scale: 0.083<br>variance prior df: 1.15

For manuscripts utilizing custom algorithms or software that are central to the research but not yet described in published literature, software must be made available to editors and reviewers. We strongly encourage code deposition in a community repository (e.g. GitHub). See the Nature Portfolio guidelines for submitting code & software for further information.

Data

Policy information about availability of data

All manuscripts must include a data availability statement. This statement should provide the following information, where applicable:

- Accession codes, unique identifiers, or web links for publicly available datasets
- A description of any restrictions on data availability
- For clinical datasets or third party data, please ensure that the statement adheres to our policy

Mass spectrometry data have been deposited to the ProteomeXchange Consortium via the PRIDE partner repository with the dataset identifier PXD PXD026654. Database Uniprot_UP000005604_Hsapiens_20191107 was used to identify proteins.Source data are provided with this paper. Uncropped scans of all blots and gels generated in this study are provided in the Source Data file. Data presented in graphs are provided in the Source Data file.

Human research participants

Policy information about studies involving human research participants and Sex and Gender in Research.

Reporting on sex and gender	N/A
Population characteristics	N/A
Recruitment	N/A
Ethics oversight	N/A

Note that full information on the approval of the study protocol must also be provided in the manuscript.

Field-specific reporting

Please select the one below that is the best fit for your research. If you are not sure, read the appropriate sections before making your selection.

☒ Life sciences ☐ Behavioural & social sciences ☐ Ecological, evolutionary & environmental sciences

For a reference copy of the document with all sections, see nature.com/documents/nr-reporting-summary-flat.pdf

Life sciences study design

All studies must disclose on these points even when the disclosure is negative.

Sample size	No statistical methods were used to pre-determine sample sizes, which were chosen based on previous experience with these type of experiments. For quantified results, the number of replicates is indicated in the figure legends.
Data exclusions	No data was excluded from this study.
Replication	Figures display representative results from one independent experiment. 5-azadC sensitivity: 4 technical replicates were used for each biological replicate. Figures show average data of 3 biological repeats. Cell viability: at least 3 biological repetitions were performed in this study. 3 technical replicates were used for each experiment. Figures display representative results from one independent experiment. All other experiments were performed as two or more independent experiments. The same conclusions were obtained from each experiment.
Randomization	In each experiment, cells were processed together and treatments were randomly allocated.
Blinding	Blinding was not relevant for our study because our analyses were analyst independent.

Reporting for specific materials, systems and methods

We require information from authors about some types of materials, experimental systems and methods used in many studies. Here, indicate whether each material, system or method listed is relevant to your study. If you are not sure if a list item applies to your research, read the appropriate section before selecting a response.

Materials & experimental systems

n/a	Involved in the study
☐	☒ Antibodies
☐	☒ Eukaryotic cell lines
☒	☐ Palaeontology and archaeology
☒	☐ Animals and other organisms
☒	☐ Clinical data
☒	☐ Dual use research of concern

Methods

n/a	Involved in the study
☒	☐ ChIP-seq
☐	☒ Flow cytometry
☒	☐ MRI-based neuroimaging

Antibodies

Antibodies used

Anti-DNMT1 (D63A6) antibody (1:1000) (#5032, Cell Signaling)
Anti-Actin antibody (1:1000) (Sc-47778, Santa Cruz Biotechnology)
Anti-SUMO2/3 antibody (1:2000) (ab3742, Abcam)
Anti-TOP1 antibody (1:1000) (ab109374, Abcam)
Anti-Ub antibody (1:1000) (Sc-8017, Santa Cruz Biotechnology)
Anti-SPRTN antibody (1:500) (6F2) Stingele lab, Zhao et al 2021
Anti-RNF4 antibody (1:500) (AF7964, R&D systems)
Anti-GAPDH (1:2000) (14C10) antibody (2118, Cell Signaling)
Anti-Histone H2A antibody (1:1000) (07-146, Merck)
Anti-Histone H2B antibody (1:1000) (12364, Cell Signaling)
Anti-Histone H3 antibody (1:1000) (4499S, Cell Signaling)
Anti-PIAS4 antibody (1:500) (SC-166744, Santa Cruz Biotechnology)
Anti-Flag (1:2000) (F1804, Sigma-Aldrich)
Anti-TEX264 (1:500) (sc-100944, Santa Cruz Biotechnology)
Anti-Vinculin (1:1000) (sc-73614, Santa Cruz.).

Validation

All commercial antibodies were validated by suppliers and/or prior references. Antibodies were additionally validated further by siRNA or knock-out cells, as indicated below:

Anti-DNMT1 (D63A6) antibody (#5032, Cell Signaling): https://www.cellsignal.de/products/primary-antibodies/dnmt1-d63a6-xp-rabbit-mab/5032?Ntk=Products, further validated by DNMT1 overexpression, Knock-down and knock-out pool
Anti-Actin antibody (Sc-47778, Santa Cruz Biotechnology): https://www.scbt.com/p/beta-actin-antibody-c4
Anti-SUMO2/3 antibody (ab3742, Abcam): https://www.abcam.com/sumo-2--sumo-3-antibody-ab3742.html, validated by UBC9 knock-down and chemical inhibition of the sumo activating enzyme with ML-792
Anti-TOP1 antibody (ab109374, Abcam): https://www.abcam.com/topoisomerase-i-antibody-epr5375-ab109374.html, validated by knock-down
Anti-Ub antibody (Sc-8017, Santa Cruz Biotechnology): https://www.scbt.com/p/ubiquitin-antibody-p4d1, validated by chemical inhibition of the ubiquitin activating enzyme with TAK-243
Anti-SPRTN antibody (6F2): Stingele lab, Zhao et al 2021, validated by siRNA
Anti-RNF4 antibody (AF7964, R&D systems): https://www.rndsystems.com/products/human-rnf4-antibody_af7964, further validated by siRNA and knock-out cells
Anti-GAPDH (14C10) antibody (2118, Cell Signaling): https://www.cellsignal.de/products/primary-antibodies/gapdh-14c10-rabbit-mab/2118
Anti-Histone H2A antibody (07-146, Merck): https://www.merckmillipore.com/DE/de/product/Anti-Histone-H2A-acidic-patch-Antibody,MM_NF-07-146
Anti-Histone H2B antibody (12364, Cell Signaling): https://www.cellsignal.de/products/primary-antibodies/histone-h2b-d2h6-rabbit-mab/12364
Anti-Histone H3 antibody (4499S, Cell Signaling): https://www.cellsignal.de/products/primary-antibodies/histone-h3-d1h2-xp-rabbit-mab/4499
Anti-PIAS4 antibody (SC-166744, Santa Cruz Biotechnology): https://www.scbt.com/p/piasy-antibody-f-9, further validated by siRNA and knock-out cells
Anti-Flag (F1804, Sigma-Aldrich): https://www.sigmaaldrich.com/DE/de/product/sigma/f1804, validated by expression of constructs carrying the Flag epitope
Anti-TEX264 (sc-100944, Santa Cruz Biotechnology): https://www.scbt.com/p/tex264-antibody-137-1, further validated by knock-out cells
Anti-Vinculin (sc-73614, Santa Cruz): https://www.scbt.com/p/vinculin-antibody-7f9

Eukaryotic cell lines

Policy information about cell lines and Sex and Gender in Research

Cell line source(s)	HeLa, U2OS T-REx Flp-In and HeLa T-REx Flp-In cells were provided by Cell Services, The Francis Crick Institute.
Authentication	The cells used in this study were originally obtained from The Francis Crick Institute and not further validated.
Mycoplasma contamination	All cells tested negative for mycoplasma contamination with the Mycoplasma PCR Detetion Kit (abm, G238).

Commonly misidentified lines (See ICLAC register)	No commonly misidentified cell lines were used in this study.

Flow Cytometry

Plots

Confirm that:

☒ The axis labels state the marker and fluorochrome used (e.g. CD4-FITC).

☒ The axis scales are clearly visible. Include numbers along axes only for bottom left plot of group (a 'group' is an analysis of identical markers).

☒ All plots are contour plots with outliers or pseudocolor plots.

☒ A numerical value for number of cells or percentage (with statistics) is provided.

Methodology

Sample preparation	Human U2OS T-REx FlpIn, HeLa or HeLa T-REx FlpIn cells were treated, washed with PBS and stained with eFluor780 viability dye and then fixed in 4% formaldehyde. Cells were then subjected to click-chemistry, adding a Alexa Fluor 488-Azide fluorophore to a previously incorporated 5-Ethynyl-2'-Desoxyuridin/5-Ethynyl-2'-uridin. At least 10,000 cells per conditions were recorded.
Instrument	BD LSRFortessa (BD Bioscience), Model number: 647800E6
Software	FlowJo™ v10.8.1 Software (BD Life Sciences)
Cell population abundance	At least 70-80% of cells were of relevant population (alive, single cells)
Gating strategy	Initial gates-SSC-A/FSC-A to discriminate cells from debris; then cells were gated SSC-H/SSC-A to discriminate single cells; then cells were gated SSC-A/APC-Cy7-A to discriminate live cells, which were then analyzed for fluorescence intensity (FITC-A, Alexa Fluor 488).

☒ Tick this box to confirm that a figure exemplifying the gating strategy is provided in the Supplementary Information.
